# Supplementary material for: Why do acute healthcare staff engage in unprofessional behaviours towards each other and how can these behaviours be reduced? A realist review protocol
Source: BMJ Open. 2022 Jul 4;12(7):e061771. doi: 10.1136/bmjopen-2022-061771 (PMC9255388; doi:10.1136/bmjopen-2022-061771)
Supplement: Supplementary data [file bmjopen-2022-061771supp001.pdf]

Literature search strategy examples

Unprofessional Behaviours among Acute Care staff – MEDLINE systematic strategy (draft)

1. Ovid MEDLINE(R) ALL <1946 to February 01, 2022>

1 emergency medical services/ or advanced trauma life support care/ or call centers/ or emergency medical dispatch/ or emergency medical service communication systems/ 47376

2 exp airway management/ or exp emergency treatment/ or exp ambulatory care/ or exp critical care/ or exp perioperative care/ or exp preoperative care/ 456732

3 exp emergency service, hospital/ or emergency services, psychiatric/ or hotlines/ or poison control centers/ or exp "transportation of patients"/ or triage/ or critical care nursing/ or emergency nursing/ 129117

4 (emergenc* adj5 (care or service* or health* or ill* or treat* or medic* or unit* or centre* or centre* or department* or setting*)).tw,kf. 200510

5 (acute* adj5 (care or service* or health* or ill* or treat* or medic* or unit* or centre* or centre* or department* or setting* or ward?)).tw,kf. 220374

6 (trauma* adj5 (care or service* or ill* or unit* or centre* or centre* or department*)).tw,kf. 26629

7 (ambula* adj5 (care or service* or unit* or centre* or centre* or department* or setting*)).tw,kf. 26105

8 (critical* adj2 (care or ill*)).tw,kf. 92228

9 (urgent adj3 (care or service* or medic*)).tw,kf. 5298

10 "intensive care".tw,kf. 171169

11 or/1-10 [Acute Care or Ambulance services] 1058529

12 agonistic behavior/ 1778

13 exp bullying/ 5615

14 problem behavior/ 3232

15 exp harassment, non-sexual/ 5697

16 sexual harassment/ 2020

17 incivility/ 232

18 Professional Misconduct/ 3432

19 Hostility/ 5396

20 Social Discrimination/ 1494

21 bully*.tw,kf. 7035

22 harass*.tw,kf. 4646

23 intimidat*.tw,kf. 1506

24 (lateral* adj2 violence).tw,kf. 100

25 (horizontal* adj2 violence).tw,kf. 146

26 (transgressive adj3 behavio?r*).tw,kf. 39

27 (disruptive adj3 behavio?r*).tw,kf. 4362

28 (unprofessional adj3 behavio?r*).tw,kf. 399

29 (micro-aggress* or microaggress*).tw,kf. 474

30 incivil*.tw,kf. 683

31 uncivil*.tw,kf. 212

32 rude*.tw,kf. 1193

33 mistreat*.tw,kf. 2441

34 (professional* adj3 misconduct).tw,kf. 303

35 mobbing.tw,kf. 416

36 (negative behavio* or negative act?).tw,kf. 1713

37 or/12-36 [Unprofessional behaviours] 40104

38 exp Health Personnel/ 571903

39 exp Students, Health Occupations/ 79772

40 exp education, graduate/ or "internship and residency"/ or teaching rounds/ 94345

41 exp Interprofessional Relations/ 71796

42 Workplace/ 26778

43 (nurs* or midwif* or midwiv*).tw,kf. 516310

44 paramedic?.tw,kf. 6034

45 (doctor? or physician? or clinician? or surgeon? or consultant?).tw,kf. 1002988

46 (student? adj2 (medic* or health* or clinic*)).tw,kf. 65732

47 intern?.tw,kf. 11160

48 resident?.tw,kf. 178976

49 (Therapist? or Pharmacist? or Optometrist? or Nutritionist? or Dentist? or Physiotherapist?).tw,kf. 132472

50 (Audiologist? or Anatomist? or Allergist? or An?esthetist? or An?esthesiologist? or Cardiologist? or Endocrinologist? or Gastroenterologist? or GP? or Geriatrician? or Hospitalist? or Oncologist?).tw,kf. 225380

51 (Ophthalmologist? or Otolaryngologist? or Pathologist? or P?ediatrician? or Physiatrist? or Psychiatrist? or Pulmonologist? or Radiographer? or Radiologist?).tw,kf. 170433

52 medic?.tw,kf. 21462

53 assistant?.tw,kf. 28781

54 (cleaner? or ancillary or porter?).tw,kf. 24901

55 (auxillary or auxillaries or administrator? or secretary or secretaries or receptionist? or technician?).tw,kf. 41498

56 (employee? or worker? or Staff or personnel or practitioner? or professional? or workforce*).tw,kf. 917727

57 (workplace* or "work place*" or worksite* or "work site*" or "work setting*").tw,kf. 55952

58 or/38-57 [Staff] 2959526

59 37 and 58 [UB and Staff search 1] 14614

60 physician-nurse relations/ or interprofessional relations/ 54899

61 aggression/ or prejudice/ or ageism/ or gender equity/ or homophobia/ or racism/ or sexism/ or weight prejudice/ or xenophobia/ 69260

62 60 and 61 [Aggression or prejudice among staff MeSH] 740

63 ((staff or employee* or work* or nurs* or doctor?) adj8 (WPV or violen*) adj5 among*).tw,kf. 560

64 ((staff or employee* or work* or nurs* or doctor?) adj8 Victim* adj5 among*).tw,kf. 109

65 ((staff or employee* or work* or nurs* or doctor?) adj8 hostil*).tw,kf. 497

66 ((staff or employee* or work* or nurs* or doctor?) adj3 undermin*).tw,kf. 334

67 ((staff or employee* or work* or nurs* or doctor?) adj6 discriminat*).tw,kf. 3201

68 "abusive supervision".tw,kf. 140

69 (workplace adj3 conflict*).tw,kf.174

70 ((staff or employee* or work* or nurs* or doctor?) adj5 (gender adj2 (inequalit* or equalit* or discriminat*))).tw,kf. 281

71 ((staff or employee* or work* or nurs* or doctor?) adj5 (racism or racist or (racial adj3 abus*))).tw,kf. 330

72 ((staff or employee* or work* or nurs* or doctor?) adj5 (sexism or sexist)).tw,kf. 83

73 ((staff or employee* or work* or nurs* or doctor?) adj8 ((disabilit* or disabled) adj5 (inequalit* or equalit* or discriminat*))).tw,kf. 97

74 ((staff or employee* or work* or nurs* or doctor?) adj8 ableis*).tw,kf. 6

75 or/62-74 [UB and staff search 2]6234

76 59 or 75 [UB among Staff final search] 20009

77 11 and 76 [UB among Staff in Acute Care] 960

78 afghanistan/ or africa/ or africa, northern/ or africa, central/ or africa, eastern/ or "africa south of the sahara"/ or africa, southern/ or africa, western/ or albania/ or algeria/ or andorra/ or angola/ or "antigua and barbuda"/ or argentina/ or armenia/ or azerbaijan/ or bahamas/ or bahrain/ or bangladesh/ or barbados/ or belize/ or benin/ or bhutan/ or bolivia/ or borneo/ or "bosnia and herzegovina"/ or botswana/ or brazil/ or brunei/ or bulgaria/ or burkina faso/ or burundi/ or cabo verde/ or cambodia/ or cameroon/ or central african republic/ or chad/ or exp china/ or comoros/ or congo/ or cote d'ivoire/ or croatia/ or cuba/ or "democratic republic of the congo"/ or cyprus/ or djibouti/ or dominica/ or dominican republic/ or ecuador/ or egypt/ or el salvador/ or equatorial guinea/ or eritrea/ or eswatini/ or ethiopia/ or fiji/ or gabon/ or gambia/ or "georgia (republic)"/ or ghana/ or grenada/ or guatemala/ or guinea/ or guinea-bissau/ or guyana/ or haiti/ or honduras/ or independent state of samoa/ or exp india/ or indian ocean islands/ or indochina/ or indonesia/ or iran/ or iraq/ or jamaica/ or jordan/ or kazakhstan/ or kenya/ or kosovo/ or kuwait/ or kyrgyzstan/ or laos/ or lebanon/ or liechtenstein/ or lesotho/ or liberia/ or libya/ or madagascar/ or malaysia/ or malawi/ or mali/ or malta/ or mauritania/ or mauritius/ or mekong valley/ or melanesia/ or micronesia/ or monaco/ or mongolia/ or montenegro/ or morocco/ or mozambique/ or myanmar/ or namibia/ or nepal/ or nicaragua/ or niger/ or nigeria/ or oman/ or pakistan/ or palau/ or exp panama/ or papua new guinea/ or paraguay/ or peru/ or philippines/ or qatar/ or "republic of belarus"/ or "republic of north macedonia"/ or romania/ or exp russia/ or rwanda/ or "saint kitts and nevis"/ or saint lucia/ or "saint vincent and the grenadines"/ or "sao tome and principe"/ or saudi arabia/ or serbia/ or sierra leone/ or senegal/ or seychelles/ or singapore/ or somalia/ or south africa/ or south sudan/ or sri lanka/ or sudan/ or suriname/ or syria/ or taiwan/ or tajikistan/ or tanzania/ or thailand/ or timor-leste/ or togo/ or tonga/ or "trinidad and tobago"/ or tunisia/ or turkmenistan/ or uganda/ or ukraine/ or united arab emirates/ or uruguay/ or uzbekistan/ or vanuatu/ or venezuela/ or vietnam/ or west indies/ or yemen/ or zambia/ or zimbabwe/ 1196785

79 "Organisation for Economic Co-Operation and Development"/ 413

80 australasia/ or exp australia/ or austria/ or baltic states/ or belgium/ or exp canada/ or chile/ or colombia/ or costa rica/ or czech republic/ or exp denmark/ or estonia/ or europe/ or finland/ or exp france/ or exp germany/ or greece/ or hungary/ or iceland/ or ireland/ or israel/ or exp italy/ or exp japan/ or korea/ or latvia/ or lithuania/ or luxembourg/ or mexico/ or netherlands/ or new zealand/ or north america/ or exp norway/ or poland/ or portugal/ or exp "republic of korea"/ or "scandinavian and nordic countries"/ or slovakia/ or slovenia/ or spain/ or sweden/ or switzerland/ or turkey/ or exp united kingdom/ or exp united states/ 3378869

81 European Union/ 17082

82 Developed Countries/ 21055

83 or/79-82 3394093

84 78 not 83 [OECD search filter NICE 2021] 1110107

85 77 not 84 [UB among Acute Care Staff with non-OECD countries removed] 872

86 (exp Child/ or Adolescent/ or exp Infant/) not exp Adult/2018609

87 85 not 86 [Child studies removed] 804

88	(elder mistreat* or elder abuse* or elder neglect*).tw,kf.	1880
89	87 not 88 [Elder abuse studies removed]	780

Google Scholar search strategy examples

Google Scholar - Theories
Search 1
bullying | harassment | discrimination|unprofessional AND workplace|worker|staff AND model|framework|concept|idea|opinion|theory|view|perception|attitude|theories [Searched in Publish or Perish Title Field]

Search 2 – Interventions & strategies
interventions|strategies|techniques|program|programs|programme|programmes AND unprofessional|bullying|harassment|discrimination AND nurse|doctor|paramedic|hospital|ambulance|staff|professional AND emergency|acute|trauma [searched in Publish or Perish Keywords field]
