# Supplementary material for: Why do acute healthcare staff engage in unprofessional behaviours towards each other and how can these behaviours be reduced? A realist review protocol
Source: BMJ Open. 2022 Jul 4;12(7):e061771. doi: 10.1136/bmjopen-2022-061771 (PMC9255388; doi:10.1136/bmjopen-2022-061771)
Supplement: Author's manuscript [file bmjopen-2022-061771.draft_revisions.pdf]

BMJ Open is committed to open peer review. As part of this commitment we make the peer review history of every article we publish publicly available.

When an article is published we post the peer reviewers' comments and the authors' responses online. We also post the versions of the paper that were used during peer review. These are the versions that the peer review comments apply to.

The versions of the paper that follow are the versions that were submitted during the peer review process. They are not the versions of record or the final published versions. They should not be cited or distributed as the published version of this manuscript.

BMJ Open is an open access journal and the full, final, typeset and author-corrected version of record of the manuscript is available on our site with no access controls, subscription charges or pay-per-view fees (http://bmjopen.bmj.com).

If you have any questions on BMJ Open's open peer review process please email info.bmjopen@bmj.com

BMJ Open

Strategies to address unprofessional behaviours among staff in acute healthcare settings and their causes: a realist review protocol

Journal:	*BMJ Open*
Manuscript ID	bmjopen-2022-061771
Article Type:	Protocol
Date Submitted by the Author:	04-Feb-2022
Complete List of Authors:	Maben, Jill; University of Surrey, School of Health Sciences<br>Aunger, Justin; University of Surrey, School of Health Sciences<br>Abrams, Ruth; University of Surrey, School of Health Sciences, Faculty of Health and Medical Sciences<br>Pearson, Mark; Hull York Medical School, Wolfson Palliative Care Research Centre<br>Wright, Judy; University of Leeds<br>Westbrook, Johanna; Macquarie University<br>Mannion, Russell; University of Birmingham, Health Services Management Centre<br>Jones, Aled; Cardiff University
Keywords:	Health & safety < HEALTH SERVICES ADMINISTRATION & MANAGEMENT, Health policy < HEALTH SERVICES ADMINISTRATION & MANAGEMENT, Quality in health care < HEALTH SERVICES ADMINISTRATION & MANAGEMENT, Organisation of health services < HEALTH SERVICES ADMINISTRATION & MANAGEMENT

SCHOLARONE™
Manuscripts

BMJ Open

Strategies to address unprofessional behaviours among staff in acute healthcare settings and their causes: a realist review protocol

Prof. Jill Maben[1,*], Dr. Justin Avery Aunger[1], Dr. Ruth Abrams[1], Dr. Mark Pearson[2], Ms. Judy M Wright[3], Prof. Johanna Westbrook[4], Prof. Russell Mannion[5], Prof. Aled Jones[6].

[*]Corresponding author

Affiliations:
[1] School of Health Sciences, Faculty of Health & Medical Sciences, University of Surrey, Kate Granger Building, Priestly Road, Surrey Research Park, Guildford, Surrey, GU2 7YH, United Kingdom
[2] Wolfson Palliative Care Research Centre, Hull York Medical School, Allam Medical Building, University of Hull, Hull, HU6 7RX, United Kingdom
[3] Leeds Institute of Health Sciences, Worsley Building, University of Leeds, Leeds, LS2 9NL, United Kingdom
[4] Centre for Health Systems and Safety Research, Australian Institute of Health Innovation, Macquarie University, Australia.
[5] Health Services Management Centre, Park House, University of Birmingham, Birmingham, B15 2RT, United Kingdom
[6] School of Healthcare Sciences, College of Biomedical and Life Sciences, Cardiff University, Eastgate House, 35-43 Newport Road, Cardiff, CF24 0AB, United Kingdom

Email addresses:
Jill Maben: J.Maben@surrey.ac.uk
Justin Avery Aunger: J.Aunger@surrey.ac.uk
Ruth Abrams: R.Abrams@surrey.ac.uk
Mark Pearson: Mark Pearson Mark.Pearson@hyms.ac.uk
Judy M Wright: J.M.Wright@Leeds.ac.uk
Johanna Westbrook: johanna.westbrook@mq.edu.au
Russell Mannion: r.mannion@bham.ac.uk
Aled Jones: JonesA97@cardiff.ac.uk

Word count: 4145

For peer review only

Abstract

Introduction

Unprofessional behaviours encompass many behaviours including bullying, harassment, and microaggressions. These behaviours between healthcare staff are problematic; they affect people's ability to work well, to feel psychologically safe at work and speak up, and to deliver safe care to patients. Almost a fifth of UK NHS staff experience unprofessional behaviours in the workplace, with higher incidence in acute care settings and for staff from minority backgrounds. Existing analyses have investigated the effectiveness of strategies at reducing these behaviours. We seek to build upon these, but go further, to understand the range and causes of such behaviours, their negative effects, and how and why mitigation strategies may work, or not, in specific contexts, and for whom.

For peer review only - http://bmjopen.bmj.com/site/about/guidelines.xhtml

BMJ Open

Methods and analysis

This study uses a realist review methodology with stakeholder input comprising a number of iterative steps: (1) formulating initial programme theories drawing on informal literature searches and literature already known to the study team, (2) performing systematic and purposive searches for grey and peer-reviewed literature, (3) selecting appropriate documents while considering rigour and relevance, (4) extracting data, (5) and synthesising and (6) refining the programme theories by testing the theories against the newly identified literature.

Ethics and dissemination

Ethical review is not required as this study is secondary research. An impact strategy has been developed which includes working closely with key stakeholders throughout the project. Step 7 of our project will be to develop pragmatic resources for managers and other professionals, tailoring contextually-sensitive strategies to reduce unprofessional behaviours, identifying what works for which groups. We will also be guided by the 'Evidence Integration Triangle' to implement the best strategies to reduce unprofessional behaviours in given contexts. Dissemination will occur through presentation at conferences, innovative methods (cartoons, videos, animations and/or interactive performances), and peer-reviewed journals.

PROSPERO Registration:

This review is registered at PROSPERO with ID CRD42021255490.

Keywords:

Incivility; bullying; professionalism; psychological safety; workforce

Article summary

Strengths and Limitations

- This review is the first to include a wide range of unprofessional behaviours among staff (not only bullying and harassment) and identify their causes for a range of staff groups and contexts in practice.
- The review utilises a context-sensitive realist methodology that accounts for complexity, to understand how and why strategies to reduce staff-to-staff unprofessional behaviours in acute care settings work and for whom.
- Realist methods allow exploration of not just 'if' an intervention or strategy works, but *how* it works, for which groups of staff and in which contexts.
- This review includes stakeholder engagement throughout, including refinement of programme theory and co-creation of actionable, context-specific outputs.
- Limitations of the review include a primary focus on acute healthcare settings; the inclusion of sources published only in English and those with similarities to a UK healthcare context.

For peer review only

BMJ Open

Introduction

Unprofessional behaviours can negatively impact staff wellbeing, patient safety and have organisational cost implications [1,2]. They are a pervasive issue in workplaces throughout global healthcare systems [2–4]. The term unprofessional behaviours is a generic one which includes uncivil, transgressive or disruptive behaviours, physical and verbal aggression [5] and bullying [6]. Unprofessional behaviours have been defined as "*a wide spectrum that includes conduct that more subtly interferes with team functioning, such as poor or ambiguous communication, passive aggression, lack of responsiveness, public criticism of colleagues, and humour at others' expense*" [7]. As such, unprofessional behaviours can be casual and generalised or highly targeted with the intention to cause harm.

Moreover, unprofessional behaviours aren't just 'unprofessional' but are increasingly being recognised as unacceptable in a modern, inclusive health setting, especially as they detrimentally affect the work and psychological wellbeing of others. These behaviours can target women and members of minority communities specifically through micro-aggressions [6,8] and, for example, misogynist and racist harassment [9–11]. Unprofessional behaviours can vary in terms of intensity and pervasiveness and remain prevalent in healthcare workplaces in the UK and worldwide [2,7,12]. This review seeks to identify the range of complex unprofessional behaviours of healthcare staff towards each other, the causes of these behaviours, their negative effects, and how, why, and for whom strategies designed to reduce unprofessional behaviours work in different contexts.

Prevalence of unprofessional behaviours in the UK healthcare

Workplace bullying affects staff in all roles and at all levels throughout the UK healthcare system. The 2020 UK National Health Service (NHS) staff survey found that 18.7% of all staff reported experiencing bullying or harassment at work from their colleagues [12], and 12.4% of staff reported experiencing these behaviours from managers [12]. Staff in ambulance service trusts and certain minority groups experience above average rates of bullying and harassment [13]. For example, the 2020 staff survey showed that minority ethnic respondents reported 5.6% higher rates of bullying and harassment from other staff than did white respondents [12].

Impacts of unprofessional behaviours

Unprofessional behaviours can have negative impacts on staff psychological wellbeing in the workplace, on patient safety and quality of care, and for wider systems and culture [14–17]. For example, bullying of individual staff members in the healthcare workplace can lead to psychological issues such as depression, burnout, and distress [18]. Bullying can also lead to physical problems such as sleep disturbance, headache, and gastrointestinal upset, resulting in staff taking sick leave [2]. In extreme cases, bullying and harassment can lead to suicidal ideation [19].

Unprofessional behaviours can result in staff feeling unsafe at work and an absence of psychological safety may encourage a climate of unprofessional behaviours and inhibit speaking up [1]. Psychological safety at work is absent in teams where incivility, transgressive behaviours, and bullying and harassment are experienced [20]. This can result

For peer review only - http://bmjopen.bmj.com/site/about/guidelines.xhtml

in reduced staff psychological well-being at work and reduced job satisfaction, engagement, and motivation [21].

The impact of unprofessional behaviours on speaking up and patient safety is well documented [1,22,23]. Where a culture of psychological safety is encouraged, employees are more likely to speak up about errors in patient care [23]. Psychologically safe teams share significant information and perform better together, improving patient safety [24–26]. For example, a recent simulation study conducted in the USA with nursing students found that where there was bullying the work environment became chaotic, overwhelming, and increased the prevalence of unsafe practices [27]. Another study performed in Israel found that team-targeted rudeness alone explained 12% of the variance in team care performance with significant negative clinical outcomes [25].

Unprofessional behaviours are associated with higher staff turnover and other significant financial costs such as litigation, lower staff productivity and sickness leave [28–31]. Kline & Lewis' (2019) conservative estimate of the financial cost of bullying and harassment to the NHS (due to sickness absence, employee turnover, reduced productivity, compensation, and litigation costs) suggests damages from bullying alone was at least £2.28 billion per annum, or 1.52% of the NHS' budget for 2019/2020 [14].

Strategies to reduce unprofessional behaviours

Unprofessional behaviours, such as bullying, constitute a complex problem that require a broad-ranging, strategic approach that incorporates multiple levels of analysis [7]. One systematic review of workplace incivility in nursing for example, identified many potential strategies [32]; these included education about workplace incivility and its impacts, assertiveness training, rehearsal of responding to incivility, and teambuilding exercises. There are also policy-level initiatives, such as legislation against harassment that is currently enshrined in UK and European law [33]. A recent Cochrane review [33] identifies that strategies can operate on society/policy, organisation, job/task, and individual/job interface levels. Such strategies can also be preventative, ameliorative, or reactive, in nature [33].

To date, much of the policy focus with regard to addressing unprofessional behaviours in healthcare settings has been on identifying 'bad apples' (individual instigators), with a more recent focus on the role of the organisational environments and cultures in which they work ('bad barrels') [34]. There is also a role for healthcare professions regulators to regulate their own organisations ('bad cellars'), as well as considering the shifting wider political and policy context ('bad orchards') [34]. Capturing these different levels of analysis and interventions is a complex task, requiring a methodology capable of understanding how strategies interact to produce particular outcomes in different contexts and for whom. A realist approach is one such methodology.

Existing syntheses and our contribution

Our realist review will complement and extend other recent reviews [35,36]. These include, for example, a systematic review which identifies the predictors and triggers of staff incivility within healthcare teams [35]. That review identified many factors contributing to incivility, including high workload, communication issues, lack of support, poor leadership,

For peer review only - http://bmjopen.bmj.com/site/about/guidelines.xhtml

and being more junior in a team. Similarly, a realist-informed review of bullying and harassment concluded that getting management on board, and wider organisational commitment, was key to intervention success [36]. While this review focused solely on bullying and harassment [36], others have mapped the terrain without providing sufficient granular detail to differentiate between professional groups and contexts, or have included unprofessional behaviours towards patients [37]. They have primarily investigated the effectiveness of strategies for reducing unprofessional behaviours but do not always explain why they do or do not work.

Our realist review differs from these by focussing on unprofessional behaviours among all staff to produce knowledge to enable more granular detail about what causes such behaviours as well as improve context-specific understanding of how, why and in what circumstances unprofessional behaviours between staff in acute healthcare settings can be best understood, mitigated, managed, and prevented. The existing literature, while useful, has not explored in sufficient detail *how* causes of unprofessional behaviour may contribute to incivility in the workplace [35], the role of context, and how such causes may interact differently with strategies (and their mechanisms of action) when seeking to reduce unprofessional behaviour. Lastly, we will look beyond single professions to consider multiple professional groups and associated team-based and organisational interprofessional dynamics, which may impact on how unprofessional behaviours emerge or are addressed.

Methods

Review aim, objectives, and research questions

Aim

This realist review aims to improve context-specific understanding of how, why and in what circumstances unprofessional behaviours between staff in acute healthcare settings can be best understood, mitigated, managed and prevented.

Objectives

This review seeks to:

1. Conceptualise and refine terminology: by mapping behaviours defined as unprofessional to understand differences and similarities between terms referring to unprofessional behaviours (e.g., incivility, bullying, micro-aggressions, etc.) and how these terms are used by different professional groups in acute healthcare settings
2. Develop and refine Context, Mechanism and Outcome configurations (CMOCs): to understand the causes and contexts of unprofessional behaviours; the mechanisms which trigger different behaviours; and the outcomes on staff, patients, and wider system of healthcare
3. Identify how, why, and for whom strategies work in different contexts: by identifying strategies designed to mitigate, manage, and prevent unprofessional behaviours and exploring how, why and in what circumstances these are most useful.

For peer review only

4. Produce recommendations and comprehensive resources that support the tailoring, implementation, monitoring and evaluation of contextually-sensitive strategies to tackle unprofessional behaviours and their impacts.

Research questions

- In what ways are unprofessional behaviours defined, developed, and experienced by staff in acute healthcare settings?
- In what ways do current strategies to reduce unprofessional behaviours in acute care address these behaviours or not?
- What are the mechanisms acting at individual, group, professional, and organisational levels that underpin strategies aimed at reducing unprofessional behaviours?
- What are the outcomes of unprofessional behaviours on staff (well-being; psychological safety), organisations (recruitment, turnover) and patients (e.g., patient safety and care quality)?
- What are the contexts which determine whether the different mechanisms produce their intended outcomes?
- What changes are needed to existing and/or future strategies to make them more effective?

Study design

This study uses a realist review methodology. Realist reviews are theory-driven and synthesise literature about complex social interventions [38]. They focus on understanding the mechanisms by which strategies work (or not) and seek to understand contextual influences on whether, why, how and for whom these might work [39]. In a realist understanding, contexts are either 1) observable features (space, place, people, things) that trigger or block an intervention with activation on a continuum, rather than a binary trigger (on/off switch) [40] or 2) relational and dynamic features that shaped the mechanisms through which an intervention works [41]. Likewise, mechanisms are interpreted as changes to participant reasoning in response to resources (e.g., education about unprofessional behaviours) introduced by an intervention [40].

The realist approach to data collection and analysis is driven by retroduction, a form of logical inference, which starts with the empirical, and explains outcomes and events by identifying the underlying mechanisms which are capable of producing them [42]. It is therefore essential to consider not only the specific unprofessional behaviours within the healthcare workforce, but any differences and similarities between staff groups as well (e.g., by specialty, professional group, setting, seniority). By illuminating these contexts and working practices, we will also be able to determine how they might influence the presence or minimisation of unprofessional behaviours between and within healthcare staff working in acute settings.

This review will follow the Realist and Meta-Review Evidence Synthesis: Evolving Standards (RAMESES) guidelines on quality and reporting [43]. An overview of the review process is

For peer review only - http://bmjopen.bmj.com/site/about/guidelines.xhtml

BMJ Open

shown in Figure 1, with the steps we will take to achieve our objectives explained in the following sections.

For peer review only

For peer review only - http://bmjopen.bmj.com/site/about/guidelines.xhtml

BMJ Open

[Figure 1 Location]

Figure 1. Review flow diagram. Dotted line reflects elements that may potentially happen.

For peer review only

Public, patient, and stakeholder involvement

We have worked closely with a stakeholder group in formulation of the proposal and have built in engagement as a key component to inform theory development. These stakeholders come from a range of relevant backgrounds, including patients and the public, heads of professional standards bodies, members of regulatory bodies and unions in the UK, influential theorists in the field, healthcare professionals with lived experience of unprofessional behaviours, and those with minority ethnic perspectives. This protocol incorporates four timepoints where stakeholder feedback will be incorporated (figure 1). This includes sense-checking our initial programme theories, aiding in identifying the most relevant evidence, ensuring our CMOCs reflect lived experience, and refining the final programme theories. In line with the ACTIVE (**A**uthors and **C**onsumers **T**ogether **I**mpacting on e**V**idencE) framework for stakeholder involvement in systematic reviews, our methodology is analogous to a continuous, multiple-time closed event approach with stakeholders able to influence the results of the review [44].

Stakeholder group feedback will be incorporated using the following processes to ensure rigour and relevance: 1) we will record what and how aspects of the developing theory are presented to stakeholders for refinement; 2) we will record where any alterations are suggested; 3) where suggestions are not aligned with what has been identified in the literature, we will perform purposive searching to sense-check recommendations; 4) any remaining discrepancies will be discussed within the team to determine majority consensus [45]. We will take detailed notes of the meetings with the aid of a project administrator and will follow up with stakeholders individually regarding specific points raised.

Step 1: Identifying existing theories

To develop initial programme theories, we will iteratively:

a) Draw on preliminary discussions within the project team, with the healthcare workforce, patients, and the public;
b) Consult with our multidisciplinary stakeholder group as outlined above;
c) Examine healthcare literature already known to the research team (namely papers, reviews and reports identified in our initial scoping review which informed the funding proposal development), informal searching of key organisational websites (e.g., King's Fund, BMA, NHS England) and use of the 26 behaviours reported by Westbrook et al. for comparison against behaviours identified in the literature [2].

This first informal screening of the literature will sensitise the team to the breadth and depth of published and unpublished literature on unprofessional behaviours within healthcare. By investigating the theoretical underpinnings of programmes, we shall map the conceptual and theoretical landscape of unprofessional behaviour causes, outcomes, and how any identified strategies and interventions are theorised to work in acute healthcare settings. Building these initial programme theories will require iterative discussions within the project team and with our stakeholders to make sense of and synthesise the different assumptions. Once the programme theories have been developed by the project team, they will be presented to the stakeholder group to integrate their feedback and to ensure practical applicability (figure 1).

For peer review only - http://bmjopen.bmj.com/site/about/guidelines.xhtml

BMJ Open

Step 2: Searching for evidence

Realist reviews typically draw on a combination of systematic searches, more informal, purposive searches, and citation tracking and reference scanning [39]. It is also common to perform more searches later in the process in an evolutionary manner to further explain certain areas which are found to be underexplored in the programme theory. Additionally, in realist reviews, what is considered evidence often differs from other reviews. For example, non-empirical studies and commentaries can be included if they illuminate the topic being studied or can otherwise contribute to the programme theory. In realist reviews, the purpose is not to necessarily to identify every item of literature that could relate to a phenomenon, but, rather, to develop and refine a comprehensive theory to explain how the intervention works, why, for whom, and in what circumstances.

Search strategy

We will take a systematic approach and conduct searches to identify literature with which to test and refine our initial theories. In this formal, primary search we will:

a) Identify studies addressing strategies to reduce unprofessional behaviours in acute healthcare settings with all healthcare staff, to shed further light on strategies to reduce unprofessional behaviours, as well as potential causes of unprofessional behaviours. Search academic databases including Medline, Embase and CINAHL and sources of trade, policy, and grey literature. Search strategies will comprise search terms, synonyms, and index terms for: Acute care AND Healthcare staff AND Unprofessional behaviours. Searches from existing similar reviews such as Iling et al. (2013) will be consulted to aid identifying relevant search terms [36]. Searches will not be limited (e.g., by date or publication) but records tagged as ‘children’, ‘animals’ or ‘elder abuse’ will be removed, and we will seek to exclude papers describing staff to patient or patient to staff unprofessional behaviours. See supplementary file 1 for an indicative example search strategy, which is not complete, as searches are likely to evolve as is typical in a realist synthesis [39].

b) Conduct a grey literature search for professional codes of conduct produced by the Royal Colleges and the literature on cases brought to the Nursing and Midwifery Council, Health Care and Professions Council, and General Medical Council for unprofessional behaviour; using databases such as Health Management Information Consortium database and websites including NHS Employers, NHS Health Education England and Google (limited to screening the first 200 results).

Search strategies will be peer-reviewed using the PRESS (Peer Review of Electronic Search Strategies) checklist [46]. All search results will be saved in reference management software. A detailed, shared spreadsheet will record all searches conducted, to ensure transparency when reporting the search activities.

Screening and inclusion criteria

We will include both peer-reviewed and grey literature which help understand how and why strategies to reduce unprofessional behaviours in acute care settings work and whom they benefit. The following inclusion criteria will be used (table 1).

For peer review only - http://bmjopen.bmj.com/site/about/guidelines.xhtml

Table 1. Inclusion criteria.

**Category**	**Criterion**
Study design	Any (including non-empirical papers/ reports).
Study setting	Acute healthcare settings - acute, critical, emergency (and potentially wider, see relevance criteria below).
Types of unprofessional behaviour	All as exhibited and experienced by healthcare staff (not patients nor patient to staff).
Types of participants	All employed staff groups including students on placements.
Types of interventions/strategies	Individual, team, organisational, and policy level interventions. Cyber-bullying and other forms of online unprofessional behaviour included if it is staff-to-staff only.
Causes of unprofessional behaviours	All.
Outcomes	Included but not limited to all sources that focused on one or more of the following aspects: staff wellbeing (stress, burnout, resilience) staff turnover, absenteeism, malpractice claims, patient complaints, magnet hospital/recruitment, patient safety (avoidable harm, errors, speaking up rates, safety incidents, improved listening/response), cost.
Language	Only sources in English will be included.

Screening of search results will be undertaken by JA in collaboration with JM and RA. A 10% random sub-sample of the citations retrieved from searching will be reviewed independently for quality control (by a second reviewer, either MP or RA). Any disagreements will be resolved by discussion between the RF, the second reviewer and JM. If disagreements remain, then a third member of the team (AJ, RM or JW) will review and any disagreements will be resolved through further review/discussion, with final decision resting with JM.

Additional searching

If we find that we require more data to develop, confirm, refute, or refine programme theory development, we will conduct additional purposive searches using CLUSTER search methods [47] to further develop any areas of the theory which require greater evidence to fully understand. These and earlier searches will follow the iterative realist search methods described by Booth et al. (2020) [48].

For peer review only - http://bmjopen.bmj.com/site/about/guidelines.xhtml

Step 3: Article selection

Decisions will be made regarding whether a source is to be included in the study or not based on a combination of relevance (based on inclusion criteria above and ability to inform programme theories) and rigour (whether the methods used to generate the relevant data are credible and trustworthy). Assessment of rigour will focus on the extent to which sources provide a detailed description of their methods and how generalisable and plausible their findings are based on those methods [39]. Classification decisions will be checked between two reviewers and discussed with the rest of the team, with disagreements resolved as described above. JA will read all included sources and finalise article selection by including documents or studies that contain data relevant to the realist analysis – i.e., those that could inform some aspect of the programme theory.

Our formal criteria for classifying the potential relevancy of sources are, by ascending order of relevancy.

Major contribution:

- Sources which contribute to the study aims and are conducted in an NHS context in acute care (major contribution); or,
- Sources which contribute to the study aims and are conducted in an NHS context; or,
- Sources which contribute to the study aims and are conducted in contexts with similarities to the NHS (e.g., universal, publicly-funded health-care systems);

Minor contribution:

- Sources conducted in non-UK health-care systems that are markedly different to the NHS (e.g., fee-for-service, private insurance scheme systems) but where the mechanisms causing or moderating unprofessional behaviours could plausibly operate in the context of those working in the NHS.
- Sources which contribute to the study aims and can clearly help to identify mechanisms which could plausibly operate in the context of the NHS (e.g., law, police, and army).

We will prioritise the most relevant papers in relation to the above list. These sources will be sorted into the above categories in a reference manager. If we are unable to develop and refine aspects of the programme theory due to scarcity in healthcare sources, we will draw on literature from outside healthcare. Following an initial random sample of sources (10%) being selected, assessed, and discussed between two reviewers to ensure that decisions for final inclusion have been made consistently, the remaining 90% of decisions regarding rigour and relevance will be made by JA. Screening will be performed using Rayyan.ai software (http://www.rayyan.ai/) [49].

Step 4: Data extraction

In keeping with a realist retroductive analysis, relevant sections of texts that have been interpreted as related to contexts, mechanisms and/or their relationships to outcomes will be coded and organised in NVivo. This coding will be both inductive (codes created to categorise data reported in included sources) and deductive (codes created in advance of data extraction and analysis as informed by the initial programme theory). Each new

For peer review only - http://bmjopen.bmj.com/site/about/guidelines.xhtml

BMJ Open

element of relevant data will be used to test and refine aspects of the programme theory, and as it is refined, included sources will be re-scrutinised to search for data relevant to the revised programme theory that may have been missed initially. The characteristics of the included sources will be extracted separately into an Excel spreadsheet to provide a descriptive overview of (but not limited to) settings, intervention, participants, and context.

As outlined above, we will start the coding and analysis process using the literature that has been deemed to make a ‘major’ i.e., most relevant contribution to the research questions to start building and refining our programme theory, while progressively focusing the review. Articles categorised as providing ‘minor’ contributions will be analysed to address aspects of the programme theory if there is no ’major’ study which sheds light on those aspects. The aim of the review will be to reach theoretical saturation in relation to the objectives, rather than to aggregate every single study that exists in the area. All study-related decisions will be documented and recorded as part of an audit trail to increase transparency and ensure consistency and rigour.

Step 5: Synthesising evidence and drawing conclusions

Our data analysis will use realist logic to make sense of the initial programme theory. Data will be interrogated at individual, team, and organisational levels to establish their relationships. This type of analysis will enable us to understand how the most relevant and important mechanisms work in different contexts, thus, allowing us to build more transferable context-mechanism-outcome configurations – these will be fully formulated by the end of this step. During the review, we will move iteratively between the analysis of particular examples from the literature, refinement of programme theory, and further iterative searching for data to test particular subsections of the programme theory (figure 1). We will also use the following strategies to make sense of the data (table 2) [50,51].

Table 2. Strategies foundational to the realist analysis.

**Strategies for synthesising evidence**
Compare and contrast sources of evidence – for example, where evidence about interventions or its mechanisms in one source allows insights into evidence about outcomes in another paper.
Reconciling of sources of evidence – where results differ in apparently similar circumstances, further investigation is appropriate in order to find explanations for why these different results occurred.
Adjudication of sources of evidence – this will enable us to make the synthesis more manageable. This includes strategies outlined in ‘article selection’ section above, i.e. dividing papers that make ‘major’ or ‘minor’ contributions to our research questions [51].
Consolidation of sources of evidence – where outcomes differ in particular contexts, an explanation can be constructed of how and why these outcomes occur differently.

Many of these processes are analogous to the identification of demi-regularities (“*semi-predictable patterns or pathways of programme functioning” across studies)*. This type of thinking will be essential to help identify underlying mechanisms that are common to different strategies [40,52]. As required, we will also identify and incorporate middle range theories (e.g., theories around, for example, groupthink and psychological safety) to enable

For peer review only - http://bmjopen.bmj.com/site/about/guidelines.xhtml

BMJ Open

us to move beyond description and understand the 'set of assumptions' lying behind the observed associations [53].

Step 6: Testing and refining, and developing resources for stakeholders

We will then test and refine our emerging evidence informed findings and programme theory with our stakeholder group. Informed by the 'Evidence Integration Triangle' [54] and stakeholder involvement at this final timepoint, we will use findings from our realist review to produce actionable evidence to support NHS managers/leaders to better understand how work environments may help or hinder unprofessional behaviours and identify what strategies work where.

Step 7: Dissemination and impact

The project will produce six major types of output in collaboration with our stakeholder group and will present some of these at our end of study project dissemination workshop (table 3).

Table 3. Dissemination and pathways to impact and who is reached [55].

Output	Description	Timescale to benefit	How will impact be achieved?	Who is reached?
**PPI / Stakeholder Engagement.**	PPI and our stakeholder representatives will be actively involved in the production of all outputs. The stakeholder group, including healthcare staff and PPI representatives, will be encouraged to think about alternative or additional approaches to dissemination.	During project and up to 6 months after.	Engaging with the stakeholder group will enable us to understand how best to reach people like them in the most well-targeted manner.	Internal stakeholder group of study and their immediate contacts.
**Media engagement strategy.**	We will identify the most appropriate way to engage with our non-Academic stakeholder groups. For example, through engagement with relevant professional bodies (e.g., British Medical Association, General Medical Council, Royal College of Nursing, Royal College of Midwives, NHS Employers etc) and through promoting our findings via alternative publication routes (e.g., Health Services Journal, Nursing Times/Standard,	During project and up to 2 years after.	Engaging with media will allow lighter touch but wider dissemination of our key messages.	Public, academics, practitioners, managers, leaders, policymakers.

For peer review only - http://bmjopen.bmj.com/site/about/guidelines.xhtml

	BMJ, The Conversation, Twitter).			
**Resources for NHS managers/leaders.**	Tailored resources for NHS managers/leaders, and organisations supporting the healthcare workforce regarding how to implement strategies to reduce unprofessional behaviours.	End of project onwards.	Effects will be visible once stakeholders are able to implement changes and evaluate the impact of those changes.	Managers, leaders.
**Plain English summaries.**	We will create plain English summaries tailored to different audiences.	End of project and up to 2 years thereafter.	This will achieve impact through knowledge transfer in the short- to medium-term (1 month-2 years).	Healthcare professionals, managers, leaders, training providers, policymakers.
**Innovative forms of communication.**	We have had positive experiences of involving film makers and using the medium of theatre to perform research findings [56].	1-5 years.	Such outputs will create a longer-lasting impression with stakeholders than other more traditional forms of dissemination.	Healthcare professionals, managers, leaders, training providers, policymakers.
**Academic outputs.**	In addition to the main project report, an overall findings paper will be submitted to a high-impact peer-reviewed journal, and conference presentations at healthcare staff well-being conferences (such as Health Services Research UK) will be pursued.	3-5 years.	Informing the agenda for debate and action in health services and in public policy more widely.	Academics, managers, policymakers.

Discussion and conclusion

Unprofessional behaviours are prevalent in the healthcare workplace and can have negative consequences for staff retention and health, quality of care, and patient safety. Interventions to reduce such behaviours exist but are often not well founded in theory and an understanding of such interventions and how and when they may work and for whom is lacking. Additionally, there is a paucity of research into the causes of unprofessional behaviours that also considers how these interact with the strategies to reduce them. Although some reviews of interventions to reduce bullying in healthcare and other workplaces exist [31,33,36], none have tried to understand different types of intervention to tackle all forms of unprofessional behaviour beyond just bullying and harassment. This project adopts a realist review methodology to identify the causes of such behaviours and assess how, and in what circumstances, interventions to reduce unprofessional behaviours in acute care settings work, why, and for whom. By working closely with our stakeholder group, we seek to develop practical guidance which will enable stakeholders and

For peer review only

For peer review only - http://bmjopen.bmj.com/site/about/guidelines.xhtml

practitioners to determine which interventions are most applicable for certain staff groups in specific contexts.

Funding statement

This project is funded by the NIHR HS&DR Programme (grant number 131606). The views and opinions expressed herein are those of the authors and do not necessarily reflect those of the HS&DR programme.

Author contributions

JM led the conceptualisation and development of the overall study design and drafting of the protocol on which this paper is based. JAA drafted the manuscript in dialogue with JM and all authors and helped to refine the communication of the study methodology. RA, MP, JMW, AJ, RM and JW all contributed to the study conceptualisation and design of the study protocol. All authors substantively revised drafts of this manuscript and approved the final version.

Competing interests

None declared.

Patient consent

Not required.

Ethical approval

The University of Surrey's Ethics committee have confirmed that ethical review is not needed for this realist review of secondary evidence.

References

1 Katz D, Blasius K, Isaak R, *et al.* Exposure to incivility hinders clinical performance in a simulated operative crisis. *BMJ Qual Saf* 2019;**28**:750–7. doi:10.1136/bmjqs-2019-009598
2 Westbrook J, Sunderland N, Li L, *et al.* The prevalence and impact of unprofessional behaviour among hospital workers: a survey in seven Australian hospitals. *Med J Aust* 2021;**214**:31–7. doi:10.5694/mja2.50849
3 Carter M, Thompson N, Crampton P, *et al.* Workplace bullying in the UK NHS: A questionnaire and interview study on prevalence, impact and barriers to reporting. *BMJ Open* 2013;**3**. doi:10.1136/bmjopen-2013-002628
4 Jones A, Blake J, Banks C, *et al.* Speaking up about Bullying and Harassment in Healthcare: Reflections Following the Introduction of an Innovative "Speak Up" Role in NHS England. In: *Connecting Healthcare Worker Well-Being, Patient Safety and Organisational Change*. Springer 2020. 145–61.
5 Felblinger DM. Bullying, incivility, and disruptive behaviors in the healthcare setting: identification, impact, and intervention. *Front Health Serv Manage* 2009;**25**:13–23.

For peer review only - http://bmjopen.bmj.com/site/about/guidelines.xhtml

For peer review only

doi:10.1097/01974520-200904000-00003
6 Cruz D, Rodriguez Y, Mastropaolo C. Perceived microaggressions in health care: A measurement study. *PLoS One* 2019;**14**:1–11. doi:10.1371/journal.pone.0211620
7 Westbrook J, Sunderland N, Atkinson V, *et al.* Endemic unprofessional behaviour in health care: the mandate for a change in approach. *Med J Aust* 2018;**209**:380–1. doi:10.5694/mja17.01261
8 Sue DW. *Microaggressions in everyday life: Race, gender, and sexual orientation*. John Wiley & Sons 2010.
9 Wild JRL, Ferguson HJM, McDermott FD, *et al.* Undermining and bullying in surgical training: A review and recommendations by the Association of Surgeons in Training. *Int J Surg* 2015;**23**:S5–9. doi:10.1016/j.ijsu.2015.09.017
10 Pisklakov S, Tilak V, Patel A, *et al.* Bullying and Aggressive Behavior among Health Care Providers: Literature Review. *Adv Anthropol* 2013;**03**:179–82. doi:10.4236/aa.2013.34024
11 Ariza-Montes A, Muniz NM, Montero-Simó MJ, *et al.* Workplace bullying among healthcare workers. *Int J Environ Res Public Health* 2013;**10**:3121–39. doi:10.3390/ijerph10083121
12 NHS England. NHS staff survey 2020. 2021. https://www.nhsstaffsurveys.com/
13 NHS Equality and Diversity Council. WRES 2018. Published Online First: 2018.https://www.england.nhs.uk/wp-content/uploads/2018/12/wres-2018-report-v1.pdf
14 Kline R, Lewis D. The price of fear: Estimating the financial cost of bullying and harassment to the NHS in England. *Public Money Manag* 2019;**39**:166–74. doi:10.1080/09540962.2018.1535044
15 Mannion R, Davies H. Raising and responding to frontline concerns in healthcare. *BMJ* 2019;**366**:1–4. doi:10.1136/bmj.l4944
16 Rosenstein AH. Nurse-physician relationships: Impact on nurse satisfaction and retention. *Am J Nurs* 2002;**102**:26–34. doi:10.1097/00000446-200206000-00040
17 NHS Employers. Tackling bullying & harassment in the NHS. Tackling bullying NHS infographic. 2019.https://www.nhsemployers.org/articles/tackling-bullying-nhs-infographic
18 Lever I, Dyball D, Greenberg N, *et al.* Health consequences of bullying in the healthcare workplace: A systematic review. *J Adv Nurs* 2019;**75**:3195–209. doi:10.1111/jan.13986
19 Nielsen MB, Indregard A-MR, Overland S. Workplace bullying and sickness absence: a systematic review and meta-analysis of the research literature. 2016.
20 Edmondson A. Psychological safety and learning behavior in work teams. 1999;**44**:350–83.https://doi.org/10.2307/2666999
21 Edmondson A. The Role of Psychological Safety. 2019. doi:10.1002/ltl.20419
22 Martinez W, Lehmann LS, Thomas EJ, *et al.* Speaking up about traditional and professionalism-related patient safety threats: A national survey of interns and residents. *BMJ Qual Saf* 2017;**26**:869–80. doi:10.1136/bmjqs-2016-006284
23 Barzallo Salazar MJ, Minkoff H, Bayya J, *et al.* Influence of surgeon behavior on trainee willingness to speak up: A randomized controlled trial. *J Am Coll Surg* 2014;**219**:1001–7. doi:10.1016/j.jamcollsurg.2014.07.933
24 Riskin A, Erez A, Foulk TA, *et al.* Rudeness and medical team performance. *Pediatrics* 2017;**139**. doi:10.1542/peds.2016-2305

For peer review only - http://bmjopen.bmj.com/site/about/guidelines.xhtml

25 Riskin A, Erez A, Foulk TA, *et al.* The impact of rudeness on medical team performance: A randomized trial. *Pediatrics* 2015;**136**:487–95. doi:10.1542/peds.2015-1385
26 Cooper WO, Spain DA, Guillamondegui O, *et al.* Association of Coworker Reports about Unprofessional Behavior by Surgeons with Surgical Complications in Their Patients. *JAMA Surg* 2019;**154**:828–34. doi:10.1001/jamasurg.2019.1738
27 Schoville R, Aebersold M. How Workplace Bullying and Incivility Impacts Patient Safety: A Qualitative Simulation Study Using BSN Students. *Clin Simul Nurs* 2020;**45**:16–23. doi:10.1016/j.ecns.2020.01.003
28 Doran N, Fox F, Rodham K, *et al.* Lost to the NHS: A mixed methods study of why GPS leave practice early in England. *Br J Gen Pract* 2016;**66**:e128–34. doi:10.3399/bjgp16X683425
29 Hogh A, Mikkelsen E, Hansen Å. Individual Consequences of Workplace Bullying/Mobbing. In: *Bullying and Harassment in the Workplace*. 2010. 107–28. doi:10.1201/ebk1439804896-7
30 Woodrow C, Guest D. A comparison of factors associated with workplace bullying in three UK Healthcare Organisations. In: *New Challenges for a Healthy Workplace in Human Services*. Rainer Hampp Verlag 2011. 87–104.
31 British Medical Association. Workplace bullying and harassment of doctors A review of recent research. 2017. file:///Users/VWC/Downloads/Bullying and harassment research review v7 WEB.pdf
32 Armstrong N. Management of Nursing Workplace Incivility in the Health Care Settings: A Systematic Review. *Work Heal Saf* 2018;**66**:403–10. doi:10.1177/2165079918771106
33 Gillen PA, Sinclair M, Kernohan WG, *et al.* Interventions for prevention of bullying in the workplace (Review). *Cochrane Database Syst Rev* 2017;:12. doi:10.1002/14651858.CD009778.pub2
34 Mannion R, Davies H, Powell M, *et al.* Healthcare scandals and the failings of doctors: Do official inquiries hold the profession to account? *J Health Organ Manag* 2019;**33**:221–40. doi:10.1108/JHOM-04-2018-0126
35 Keller S, Yule S, Zagarese V, *et al.* Predictors and triggers of incivility within healthcare teams: A systematic review of the literature. *BMJ Open* 2020;**10**:1–15. doi:10.1136/bmjopen-2019-035471
36 Illing J, Carter M, Thompson NJ, *et al.* Evidence synthesis on the occurrence, causes , management of bullying and harassing behaviours to inform decision making in the NHS. 2013;**44**:54–168.
37 Raine G, Thomas S, Rodgers M, *et al. Workplace-based interventions to promote healthy lifestyles in the NHS workforce: a rapid scoping and evidence map*. 2020. doi:10.3310/hsdr08180
38 Pawson R, Greenhalgh T, Harvey G, *et al.* Realist review - A new method of systematic review designed for complex policy interventions. *J Heal Serv Res Policy* 2005;**10**:21–34. doi:10.1258/1355819054308530
39 Wong G, Westhorp G, Pawson R, *et al.* Realist Synthesis: RAMESES Training Materials. *RAMESES Proj* 2013;:55.
40 Dalkin SM, Greenhalgh J, Jones D, *et al.* What's in a mechanism? Development of a key concept in realist evaluation. 2015;**10**:49.https://doi.org/10.1186/s13012-015-0237-x

For peer review only - http://bmjopen.bmj.com/site/about/guidelines.xhtml

BMJ Open

41 Greenhalgh J, Manzano A. Understanding ‘context’ in realist evaluation and synthesis. *Int J Soc Res Methodol* 2021;**00**:1–13. doi:10.1080/13645579.2021.1918484
42 Sayer RA. *Method in social science: A realist approach*. Psychology Press 1992.
43 Wong G, Greenhalgh T, Westhorp G, *et al.* RAMESES publication standards: realist syntheses. *BMC Med* 2013;**11**:1–14.
44 Pollock A, Campbell P, Struthers C, *et al.* Development of the ACTIVE framework to describe stakeholder involvement in systematic reviews. *J Heal Serv Res Policy* 2019;**24**:245–55. doi:10.1177/1355819619841647
45 Abrams R, Park S, Wong G, *et al.* Lost in reviews: Looking for the involvement of stakeholders, patients, public and other non-researcher contributors in realist reviews. *Res Synth Methods* 2021;**12**:239–47. doi:10.1002/jrsm.1459
46 McGowan J, Sampson M, Salzwedel DM, *et al.* PRESS Peer Review of Electronic Search Strategies: 2015 Guideline Statement. *J Clin Epidemiol* 2016;**75**:40–6. doi:10.1016/j.jclinepi.2016.01.021
47 Booth A, Harris J, Croot E, *et al.* Towards a methodology for cluster searching to provide conceptual and contextual “richness” for systematic reviews of complex interventions: case study (CLUSTER). *BMC Med Res Methodol* 2013;**13**:1–14.
48 Booth A, Briscoe S, Wright JM. The “realist search”: A systematic scoping review of current practice and reporting. *Res Synth Methods* 2020;**11**:14–35.
49 Ouzzani M, Hammady H, Fedorowicz Z, *et al.* Rayyan-a web and mobile app for systematic reviews. *Syst Rev* 2016;**5**:1–10. doi:10.1186/s13643-016-0384-4
50 Pawson R. *Evidence-based policy: a realist perspective*. sage 2006.
51 Pearson M, Chilton R, Wyatt K, *et al.* Implementing health promotion programmes in schools: A realist systematic review of research and experience in the United Kingdom. *Implement Sci* 2015;**10**:1–20. doi:10.1186/s13012-015-0338-6
52 Gilmore B, McAuliffe E, Power J, *et al.* Data Analysis and Synthesis Within a Realist Evaluation: Toward More Transparent Methodological Approaches. *Int J Qual Methods* 2019;**18**:1–11. doi:10.1177/1609406919859754
53 Merton RK. On theoretical sociology: five essays, old and new. 1967.
54 Glasgow RE, Green LW, Taylor M V., *et al.* An evidence integration triangle for aligning science with policy and practice. *Am J Prev Med* 2012;**42**:646–54. doi:10.1016/j.amepre.2012.02.016
55 Lamont T. *Making Research Matter: Steps to Impact for Health and Care Researchers*. Policy Press 2021.
56 Maben J, Taylor C, Dawson J, *et al.* A realist informed mixed-methods evaluation of Schwartz Center Rounds® in England. *Heal Serv Deliv Res* 2018;**6**:1–260. doi:10.3310/hsdr06370

For peer review only

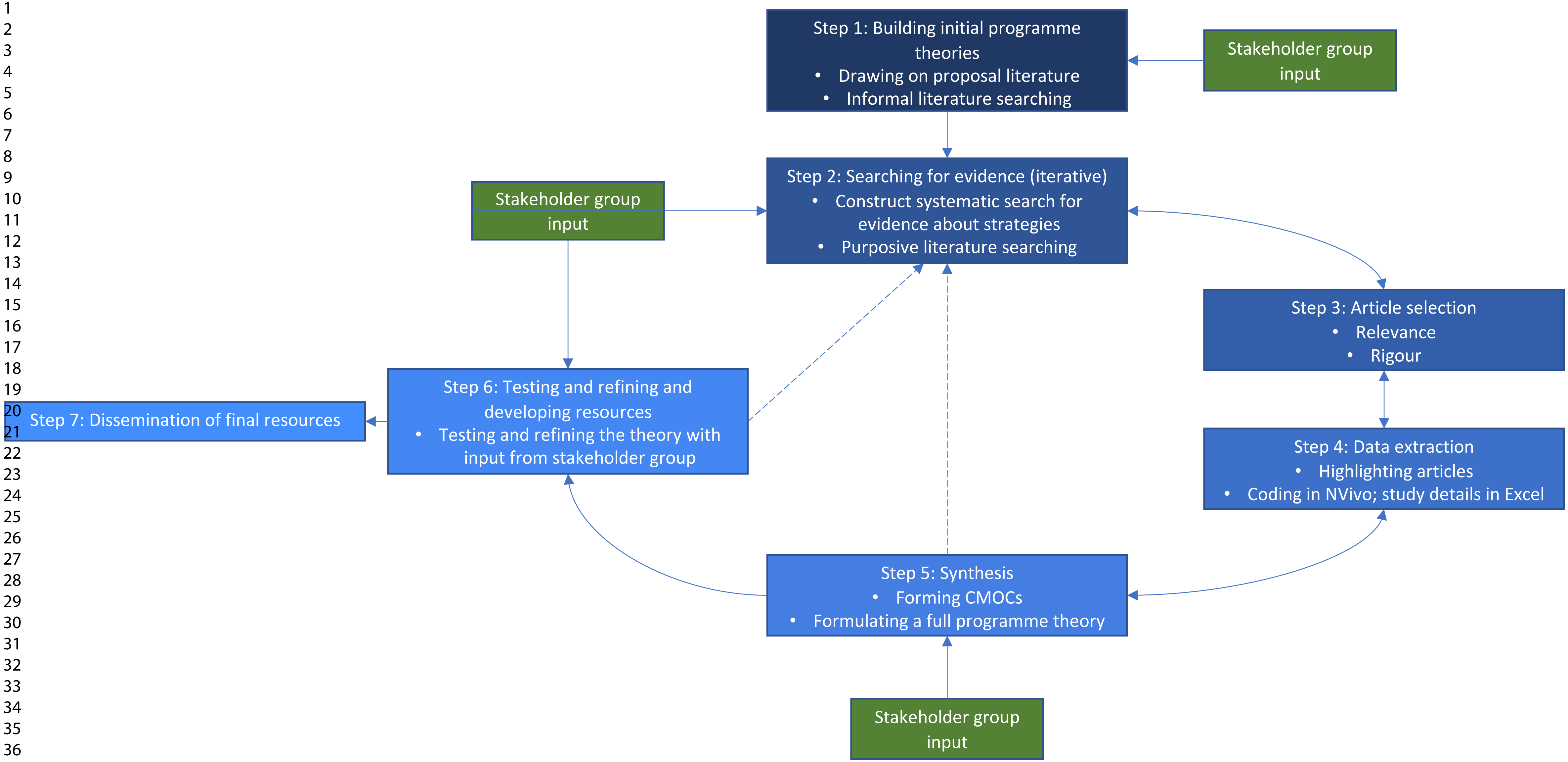
Step 1: Building initial programme theories
• Drawing on proposal literature
• Informal literature searching
Stakeholder group input
Stakeholder group input
Step 2: Searching for evidence (iterative)
• Construct systematic search for evidence about strategies
• Purposive literature searching
Step 3: Article selection
• Relevance
• Rigour
Step 7: Dissemination of final resources
Step 6: Testing and refining and developing resources
• Testing and refining the theory with input from stakeholder group
Step 4: Data extraction
• Highlighting articles
• Coding in NVivo; study details in Excel
Step 5: Synthesis
• Forming CMOCs
• Formulating a full programme theory
Stakeholder group input

Literature search strategy examples

Unprofessional Behaviours among Acute Care staff – MEDLINE systematic strategy (draft)

1. Ovid MEDLINE(R) ALL <1946 to February 01, 2022>

1 emergency medical services/ or advanced trauma life support care/ or call centers/ or emergency medical dispatch/ or emergency medical service communication systems/ 47376

2 exp airway management/ or exp emergency treatment/ or exp ambulatory care/ or exp critical care/ or exp perioperative care/ or exp preoperative care/ 456732

3 exp emergency service, hospital/ or emergency services, psychiatric/ or hotlines/ or poison control centers/ or exp "transportation of patients"/ or triage/ or critical care nursing/ or emergency nursing/ 129117

4 (emergenc* adj5 (care or service* or health* or ill* or treat* or medic* or unit* or centre* or centre* or department* or setting*)).tw,kf. 200510

5 (acute* adj5 (care or service* or health* or ill* or treat* or medic* or unit* or centre* or centre* or department* or setting* or ward?)).tw,kf. 220374

6 (trauma* adj5 (care or service* or ill* or unit* or centre* or centre* or department*)).tw,kf. 26629

7 (ambula* adj5 (care or service* or unit* or centre* or centre* or department* or setting*)).tw,kf. 26105

8 (critical* adj2 (care or ill*)).tw,kf. 92228

9 (urgent adj3 (care or service* or medic*)).tw,kf. 5298

10 "intensive care".tw,kf. 171169

11 or/1-10 [Acute Care or Ambulance services] 1058529

12 agonistic behavior/ 1778

13 exp bullying/ 5615

14 problem behavior/ 3232

15 exp harassment, non-sexual/ 5697

16 sexual harassment/ 2020

17 incivility/ 232

18 Professional Misconduct/ 3432

19 Hostility/ 5396

20 Social Discrimination/ 1494

21 bully*.tw,kf. 7035

22 harass*.tw,kf. 4646

For peer review only

23 intimidat*.tw,kf. 1506

24 (lateral* adj2 violence).tw,kf. 100

25 (horizontal* adj2 violence).tw,kf. 146

26 (transgressive adj3 behavio?r*).tw,kf. 39

27 (disruptive adj3 behavio?r*).tw,kf. 4362

28 (unprofessional adj3 behavio?r*).tw,kf. 399

29 (micro-aggress* or microaggress*).tw,kf. 474

30 incivil*.tw,kf. 683

31 uncivil*.tw,kf. 212

32 rude*.tw,kf. 1193

33 mistreat*.tw,kf. 2441

34 (professional* adj3 misconduct).tw,kf. 303

35 mobbing.tw,kf. 416

36 (negative behavio* or negative act?).tw,kf. 1713

37 or/12-36 [Unprofessional behaviours] 40104

38 exp Health Personnel/ 571903

39 exp Students, Health Occupations/ 79772

40 exp education, graduate/ or "internship and residency"/ or teaching rounds/ 94345

41 exp Interprofessional Relations/ 71796

42 Workplace/ 26778

43 (nurs* or midwif* or midwiv*).tw,kf. 516310

44 paramedic?.tw,kf. 6034

45 (doctor? or physician? or clinician? or surgeon? or consultant?).tw,kf. 1002988

46 (student? adj2 (medic* or health* or clinic*)).tw,kf. 65732

47 intern?.tw,kf. 11160

48 resident?.tw,kf. 178976

49 (Therapist? or Pharmacist? or Optometrist? or Nutritionist? or Dentist? or Physiotherapist?).tw,kf. 132472

50 (Audiologist? or Anatomist? or Allergist? or An?esthetist? or An?esthesiologist? or Cardiologist? or Endocrinologist? or Gastroenterologist? or GP? or Geriatrician? or Hospitalist? or Oncologist?).tw,kf. 225380

For peer review only

51 (Ophthalmologist? or Otolaryngologist? or Pathologist? or P?ediatrician? or Physiatrist? or Psychiatrist? or Pulmonologist? or Radiographer? or Radiologist?).tw,kf. 170433

52 medic?.tw,kf. 21462

53 assistant?.tw,kf. 28781

54 (cleaner? or ancillary or porter?).tw,kf. 24901

55 (auxillary or auxillaries or administrator? or secretary or secretaries or receptionist? or technician?).tw,kf. 41498

56 (employee? or worker? or Staff or personnel or practitioner? or professional? or workforce*).tw,kf. 917727

57 (workplace* or "work place*" or worksite* or "work site*" or "work setting*").tw,kf. 55952

58 or/38-57 [Staff] 2959526

59 37 and 58 [UB and Staff search 1] 14614

60 physician-nurse relations/ or interprofessional relations/ 54899

61 aggression/ or prejudice/ or ageism/ or gender equity/ or homophobia/ or racism/ or sexism/ or weight prejudice/ or xenophobia/ 69260

62 60 and 61 [Aggression or prejudice among staff MeSH] 740

63 ((staff or employee* or work* or nurs* or doctor?) adj8 (WPV or violen*) adj5 among*).tw,kf. 560

64 ((staff or employee* or work* or nurs* or doctor?) adj8 Victim* adj5 among*).tw,kf. 109

65 ((staff or employee* or work* or nurs* or doctor?) adj8 hostil*).tw,kf. 497

66 ((staff or employee* or work* or nurs* or doctor?) adj3 undermin*).tw,kf. 334

67 ((staff or employee* or work* or nurs* or doctor?) adj6 discriminat*).tw,kf. 3201

68 "abusive supervision".tw,kf. 140

69 (workplace adj3 conflict*).tw,kf.174

70 ((staff or employee* or work* or nurs* or doctor?) adj5 (gender adj2 (inequalit* or equalit* or discriminat*))).tw,kf. 281

71 ((staff or employee* or work* or nurs* or doctor?) adj5 (racism or racist or (racial adj3 abus*))).tw,kf. 330

72 ((staff or employee* or work* or nurs* or doctor?) adj5 (sexism or sexist)).tw,kf. 83

73 ((staff or employee* or work* or nurs* or doctor?) adj8 ((disabilit* or disabled) adj5 (inequalit* or equalit* or discriminat*))).tw,kf. 97

74 ((staff or employee* or work* or nurs* or doctor?) adj8 ableis*).tw,kf. 6

75 or/62-74 [UB and staff search 2]6234

For peer review only

76 59 or 75 [UB among Staff final search] 20009

77 11 and 76 [UB among Staff in Acute Care] 960

78 afghanistan/ or africa/ or africa, northern/ or africa, central/ or africa, eastern/ or "africa south of the sahara"/ or africa, southern/ or africa, western/ or albania/ or algeria/ or andorra/ or angola/ or "antigua and barbuda"/ or argentina/ or armenia/ or azerbaijan/ or bahamas/ or bahrain/ or bangladesh/ or barbados/ or belize/ or benin/ or bhutan/ or bolivia/ or borneo/ or "bosnia and herzegovina"/ or botswana/ or brazil/ or brunei/ or bulgaria/ or burkina faso/ or burundi/ or cabo verde/ or cambodia/ or cameroon/ or central african republic/ or chad/ or exp china/ or comoros/ or congo/ or cote d'ivoire/ or croatia/ or cuba/ or "democratic republic of the congo"/ or cyprus/ or djibouti/ or dominica/ or dominican republic/ or ecuador/ or egypt/ or el salvador/ or equatorial guinea/ or eritrea/ or eswatini/ or ethiopia/ or fiji/ or gabon/ or gambia/ or "georgia (republic)"/ or ghana/ or grenada/ or guatemala/ or guinea/ or guinea-bissau/ or guyana/ or haiti/ or honduras/ or independent state of samoa/ or exp india/ or indian ocean islands/ or indochina/ or indonesia/ or iran/ or iraq/ or jamaica/ or jordan/ or kazakhstan/ or kenya/ or kosovo/ or kuwait/ or kyrgyzstan/ or laos/ or lebanon/ or liechtenstein/ or lesotho/ or liberia/ or libya/ or madagascar/ or malaysia/ or malawi/ or mali/ or malta/ or mauritania/ or mauritius/ or mekong valley/ or melanesia/ or micronesia/ or monaco/ or mongolia/ or montenegro/ or morocco/ or mozambique/ or myanmar/ or namibia/ or nepal/ or nicaragua/ or niger/ or nigeria/ or oman/ or pakistan/ or palau/ or exp panama/ or papua new guinea/ or paraguay/ or peru/ or philippines/ or qatar/ or "republic of belarus"/ or "republic of north macedonia"/ or romania/ or exp russia/ or rwanda/ or "saint kitts and nevis"/ or saint lucia/ or "saint vincent and the grenadines"/ or "sao tome and principe"/ or saudi arabia/ or serbia/ or sierra leone/ or senegal/ or seychelles/ or singapore/ or somalia/ or south africa/ or south sudan/ or sri lanka/ or sudan/ or suriname/ or syria/ or taiwan/ or tajikistan/ or tanzania/ or thailand/ or timor-leste/ or togo/ or tonga/ or "trinidad and tobago"/ or tunisia/ or turkmenistan/ or uganda/ or ukraine/ or united arab emirates/ or uruguay/ or uzbekistan/ or vanuatu/ or venezuela/ or vietnam/ or west indies/ or yemen/ or zambia/ or zimbabwe/ 1196785

79 "Organisation for Economic Co-Operation and Development"/ 413

80 australasia/ or exp australia/ or austria/ or baltic states/ or belgium/ or exp canada/ or chile/ or colombia/ or costa rica/ or czech republic/ or exp denmark/ or estonia/ or europe/ or finland/ or exp france/ or exp germany/ or greece/ or hungary/ or iceland/ or ireland/ or israel/ or exp italy/ or exp japan/ or korea/ or latvia/ or lithuania/ or luxembourg/ or mexico/ or netherlands/ or new zealand/ or north america/ or exp norway/ or poland/ or portugal/ or exp "republic of korea"/ or "scandinavian and nordic countries"/ or slovakia/ or slovenia/ or spain/ or sweden/ or switzerland/ or turkey/ or exp united kingdom/ or exp united states/ 3378869

81 European Union/ 17082

82 Developed Countries/ 21055

83 or/79-82 3394093

84 78 not 83 [OECD search filter NICE 2021] 1110107

85 77 not 84 [UB among Acute Care Staff with non-OECD countries removed] 872

86 (exp Child/ or Adolescent/ or exp Infant/) not exp Adult/2018609

87 85 not 86 [Child studies removed] 804

For peer review only

88	(elder mistreat* or elder abuse* or elder neglect*).tw,kf.	1880
89	87 not 88 [Elder abuse studies removed]	780

Google Scholar search strategy examples

Google Scholar - Theories
Search 1
bullying | harassment | discrimination|unprofessional AND workplace|worker|staff AND model|framework|concept|idea|opinion|theory|view|perception|attitude|theories [Searched in Publish or Perish Title Field]

Search 2 – Interventions & strategies
interventions|strategies|techniques|program|programs|programme|programmes AND unprofessional|bullying|harassment|discrimination AND nurse|doctor|paramedic|hospital|ambulance|staff|professional AND emergency|acute|trauma [searched in Publish or Perish Keywords field]

For peer review only

	Reporting item	Description of item	Reported on page(s)
**Title**			
1		In the title, identify the document as a realist synthesis or review	1
**Abstract**			
2		While acknowledging publication requirements and house style, abstracts should ideally contain brief details of: the study's background, review question or objectives; search strategy; methods of selection, appraisal, analysis and synthesis of sources; main results; and implications for practice	1-2
**Introduction**			
3	Rationale for review	Explain why the review is needed and what it is likely to contribute to existing understanding of the topic area	4-5
4	Objectives and focus of review	State the objective(s) of the review and/or the review question(s). Define and provide a rationale for the focus of the review	5-6
**Methods**			

For peer review only

Reporting item		Description of item	Reported on page(s)
5	Changes in the review process	Any changes made to the review process that was initially planned should be briefly described and justified	Potential changes outlined on pg 10-12
6	Rationale for using realist synthesis	Explain why realist synthesis was considered the most appropriate method to use	4
7	Scoping the literature	Describe and justify the initial process of exploratory scoping of the literature	8
8	Searching processes	While considering specific requirements of the journal or other publication outlet, state and provide a rationale for how the iterative searching was done. Provide details on all of the sources accessed for information in the review. Where searching in electronic databases has taken place, the details should include, for example, name of database, search terms, dates of coverage and date last searched. If individuals familiar with the relevant literature and/or topic area were contacted, indicate how they were identified and selected	9-10
9	Selection and appraisal of documents	Explain how judgements were made about including and excluding data from documents, and justify these	10-11
10	Data extraction	Describe and explain which data or information were extracted from the included documents and justify this selection	11-12

For peer review only

Reporting item		Description of item	Reported on page(s)
11	Analysis and synthesis processes	Describe the analysis and synthesis processes in detail. This section should include information on the constructs analysed and describe the analytic process	12-13
**Results**			
12	Document flow diagram	Provide details on the number of documents assessed for eligibility and included in the review, with reasons for exclusion at each stage, as well as an indication of their source of origin (e.g. from searching databases, reference lists and so on). You may consider using the example templates (which are likely to need modification to suit the data) that are provided	7
13	Document characteristics	Provide information on the characteristics of the documents included in the review	n/a
14	Main findings	Present the key findings with a specific focus on theory building and testing	n/a
**Discussion**			
15	Summary of findings	Summarise the main findings, taking into account the reviews objective(s), research question(s), focus and intended audience(s)	n/a

For peer review only

	Reporting item	Description of item	Reported on page(s)
16	Strengths, limitations and future research directions	Discuss both the strengths of the review and its limitations. These should include (but need not be restricted to) (a) consideration of all the steps in the review process and (b) comment on the overall strength of evidence supporting the explanatory insights which emerged<br>The limitations identified may point to areas where further work is needed	2 & 14
17	Comparison with existing literature	Where applicable, compare and contrast the reviews findings with the existing literature (e.g. other reviews) on the same topic	2-5
18	Conclusion and recommendations	List the main implications of the findings and place these in the context of other relevant literature. If appropriate, offer recommendations for policy and practice	14
19	Funding	Provide details of funding source (if any) for the review, the role played by the funder (if any) and any conflicts of interests of the reviewers	15

For peer review only

BMJ Open

Why do acute healthcare staff engage in unprofessional behaviours towards each other and how can these behaviours be reduced? A realist review protocol

Journal:	*BMJ Open*
Manuscript ID	bmjopen-2022-061771.R1
Article Type:	Protocol
Date Submitted by the Author:	23-Apr-2022

Complete List of Authors:	Maben, Jill; University of Surrey, School of Health Sciences<br>Aunger, Justin; University of Surrey, School of Health Sciences<br>Abrams, Ruth; University of Surrey, School of Health Sciences, Faculty of Health and Medical Sciences<br>Pearson, Mark; Hull York Medical School, Wolfson Palliative Care Research Centre<br>Wright, Judy; University of Leeds<br>Westbrook, Johanna; Macquarie University<br>Mannion, Russell; University of Birmingham, Health Services Management Centre<br>Jones, Aled; Cardiff University

**Primary Subject Heading**:	Health services research
Secondary Subject Heading:	Health services research, Health policy, Nursing
Keywords:	Health & safety < HEALTH SERVICES ADMINISTRATION & MANAGEMENT, Health policy < HEALTH SERVICES ADMINISTRATION & MANAGEMENT, Quality in health care < HEALTH SERVICES ADMINISTRATION & MANAGEMENT, Organisation of health services < HEALTH SERVICES ADMINISTRATION & MANAGEMENT

SCHOLARONE™
Manuscripts

BMJ Open

Why do acute healthcare staff engage in unprofessional behaviours towards each other and how can these behaviours be reduced? A realist review protocol

Prof. Jill Maben[1,*], Dr. Justin Avery Aunger[1], Dr. Ruth Abrams[1], Dr. Mark Pearson[2], Ms. Judy M Wright[3], Prof. Johanna Westbrook[4], Prof. Russell Mannion[5], Prof. Aled Jones[6].

*Corresponding author

Affiliations:
[1] School of Health Sciences, Faculty of Health & Medical Sciences, University of Surrey, Kate Granger Building, Priestly Road, Surrey Research Park, Guildford, Surrey, GU2 7YH, United Kingdom
[2] Wolfson Palliative Care Research Centre, Hull York Medical School, Allam Medical Building, University of Hull, Hull, HU6 7RX, United Kingdom
[3] Leeds Institute of Health Sciences, Worsley Building, University of Leeds, Leeds, LS2 9NL, United Kingdom
[4] Centre for Health Systems and Safety Research, Australian Institute of Health Innovation, Macquarie University, Australia.
[5] Health Services Management Centre, Park House, University of Birmingham, Birmingham, B15 2RT, United Kingdom
[6] School of Healthcare Sciences, College of Biomedical and Life Sciences, Cardiff University, Eastgate House, 35-43 Newport Road, Cardiff, CF24 0AB, United Kingdom

Email addresses:
Jill Maben: J.Maben@surrey.ac.uk
Justin Avery Aunger: J.Aunger@surrey.ac.uk
Ruth Abrams: R.Abrams@surrey.ac.uk
Mark Pearson: Mark Pearson Mark.Pearson@hyms.ac.uk
Judy M Wright: J.M.Wright@Leeds.ac.uk
Johanna Westbrook: johanna.westbrook@mq.edu.au
Russell Mannion: r.mannion@bham.ac.uk
Aled Jones: JonesA97@cardiff.ac.uk

Word count: 4139

Abstract

Introduction

Unprofessional behaviours encompass many behaviours including bullying, harassment, and microaggressions. These behaviours between healthcare staff are problematic; they affect people's ability to work, to feel psychologically safe at work and speak up, and to deliver safe care to patients. Almost a fifth of UK NHS staff experience unprofessional behaviours in the workplace, with higher incidence in acute care settings and for staff from minority backgrounds. Existing analyses have investigated the effectiveness of strategies at reducing these behaviours. We seek to go beyond these, to understand the range and causes of such behaviours, their negative effects, and how mitigation strategies may work, in which contexts, and for whom.

For peer review only

BMJ Open

Methods and analysis

This study uses a realist review methodology with stakeholder input comprising a number of iterative steps: (1) formulating initial programme theories drawing on informal literature searches and literature already known to the study team, (2) performing systematic and purposive searches for grey and peer-reviewed literature on Embase, CINAHL, and Medline databases as well as Google and Google Scholar, (3) selecting appropriate documents while considering rigour and relevance, (4) extracting data, (5) and synthesising and (6) refining the programme theories by testing the theories against the newly identified literature.

Ethics and dissemination

Ethical review is not required as this study is secondary research. An impact strategy has been developed which includes working closely with key stakeholders throughout the project. Step 7 of our project will develop pragmatic resources for managers and professionals, tailoring contextually-sensitive strategies to reduce unprofessional behaviours, identifying what works for which groups. We will be guided by the 'Evidence Integration Triangle' to implement the best strategies to reduce unprofessional behaviours in given contexts. Dissemination will occur through presentation at conferences, innovative methods (cartoons, videos, animations and/or interactive performances), and peer-reviewed journals.

PROSPERO Registration:

This review is registered at PROSPERO with ID CRD42021255490.

Keywords:

Incivility; bullying; professionalism; psychological safety; workforce

Article summary

Strengths and Limitations

- This review is the first to identify the causes of a wide range of unprofessional behaviours (not only bullying and harassment) among staff for a range of staff groups and contexts in practice.
- The review utilises a context-sensitive realist methodology that accounts for complexity, to understand how and why strategies to reduce staff-to-staff unprofessional behaviours in acute care settings work and for whom.
- Reviewing both the causes of unprofessional behaviours and the strategies to reduce them will enable us to gain a better understanding of how strategies interact with and target the underlying mechanisms that lead to unprofessional behaviours.
- This review includes stakeholder engagement throughout, including refinement of programme theory and co-creation of actionable, context-specific outputs.
- Limitations of the review include a primary focus on acute healthcare settings, the inclusion of sources published only in English, and focus on health systems with similarities to a UK healthcare context.

For peer review only - http://bmjopen.bmj.com/site/about/guidelines.xhtml

BMJ Open

Introduction

Unprofessional behaviours can negatively impact staff wellbeing, patient safety and have organisational cost implications [1,2]. They are a pervasive issue in workplaces throughout global healthcare systems [2–4]. The term unprofessional behaviours is a generic one which includes a range of more specific behaviours such as uncivil, transgressive or disruptive behaviours, physical and verbal aggression [5] and bullying [6]. Unprofessional behaviours have been defined as "*a wide spectrum that includes conduct that more subtly interferes with team functioning, such as poor or ambiguous communication, passive aggression, lack of responsiveness, public criticism of colleagues, and humour at others' expense*" [7]. As such, unprofessional behaviours can be casual and generalised, or highly targeted with the intention to cause harm. Unprofessional behaviours are increasingly being recognised as unacceptable in a modern, inclusive health setting, as they detrimentally affect the work and psychological wellbeing of others. Unprofessional behaviours can vary in terms of intensity and pervasiveness and remain prevalent in healthcare workplaces in the UK and worldwide [2,7,8].

Prevalence of unprofessional behaviours in the UK healthcare system

Workplace bullying affects staff in all roles and at all levels throughout the UK healthcare system. The 2020 UK National Health Service (NHS) staff survey found that 18.7% of all staff reported experiencing bullying or harassment at work from their colleagues [8], and 12.4% of staff reported experiencing these behaviours from managers [8]. Staff in ambulance service trusts and certain minority groups experience above average rates of bullying and harassment [9], including women, individuals with a disability, and members of minority communities, through micro-aggressions [6,10] and harassment or discrimination [11–13]. For example, the 2020 staff survey showed that minority ethnic respondents reported 5.6% higher rates of bullying and harassment from other staff than did white respondents [8].

Impacts of unprofessional behaviours

Unprofessional behaviours can have negative impacts on staff psychological wellbeing in the workplace, on patient safety and quality of care, and for wider systems and culture [14–17]. For example, bullying of individual staff members in the healthcare workplace can lead to psychological issues such as depression, burnout, and distress [18]. Bullying can also lead to physical problems such as sleep disturbance, headache, and gastrointestinal upset, resulting in staff taking sick leave [2]. In extreme cases, bullying and harassment can lead to suicidal ideation [19].

Unprofessional behaviours can result in staff feeling unsafe at work and an absence of psychological safety may encourage a climate of unprofessional behaviours and inhibit speaking up [1]. Psychological safety at work is absent in teams where incivility, transgressive behaviours, and bullying and harassment are experienced [20]. This can result in reduced staff psychological well-being at work and reduced job satisfaction, engagement, and motivation [21].

The impact of unprofessional behaviours on speaking up and patient safety is well documented [1,22,23]. Where a culture of psychological safety is encouraged, employees are more likely to speak up about errors in patient care [23]. Psychologically safe teams

For peer review only - http://bmjopen.bmj.com/site/about/guidelines.xhtml

share significant information and perform better together, improving patient safety [24–26]. For example, a recent simulation study conducted in the USA with nursing students found that where there was bullying the work environment became chaotic, overwhelming, and increased the prevalence of unsafe practices [27]. Another study performed in Israel found that team-targeted rudeness alone explained 12% of the variance in team care performance with significant negative clinical outcomes [25].

Unprofessional behaviours are associated with higher staff turnover and other significant financial costs such as litigation, lower staff productivity and sickness leave [28–31]. Kline & Lewis' (2019) conservative estimate of the financial cost of bullying and harassment to the NHS (due to sickness absence, employee turnover, reduced productivity, compensation, and litigation costs) suggests damages from bullying alone was at least £2.28 billion per annum, or 1.52% of the NHS' budget for 2019/2020 [14].

Strategies to reduce unprofessional behaviours

Unprofessional behaviours, such as bullying, constitute a complex problem that require a broad-ranging, strategic approach that incorporates multiple levels of analysis [7]. One systematic review of workplace incivility in nursing for example, identified many potential strategies [32]; these included education about workplace incivility and its impacts, assertiveness training, rehearsal of responding to incivility, and teambuilding exercises. There are also policy-level initiatives, such as legislation against harassment that is currently enshrined in UK and European law [33]. A recent Cochrane review [33] identifies that strategies can operate on society/policy, organisation, job/task, and individual/job interface levels. Such strategies can also be preventative, ameliorative, or reactive, in nature [33].

To date, much of the policy focus with regard to addressing unprofessional behaviours in healthcare settings has been on identifying 'bad apples' (individual instigators), with a more recent focus on the role of the organisational environments and cultures in which they work ('bad barrels') [34]. There is also a role for healthcare professions regulators to regulate their own organisations ('bad cellars'), as well as considering the shifting wider political and policy context ('bad orchards') [34]. Capturing these different levels of analysis and interventions is a complex task, requiring a methodology capable of understanding how strategies interact to produce particular outcomes in different contexts and for whom. A realist approach is one such methodology.

Existing syntheses and our contribution

Our realist review will complement and extend other recent reviews [35,36]. These include, for example, a systematic review which identifies the predictors and triggers of staff incivility within healthcare teams, including high workload, communication issues, lack of support, poor leadership, and being more junior in a team [35]. Similarly, a realist-informed review of bullying and harassment concluded that the involvement of management and wider organisational commitment were key to intervention success [36]. While this review focused solely on bullying and harassment [36], others have mapped the terrain without providing sufficient granular detail to differentiate between professional groups and contexts, or have included unprofessional behaviours towards patients [37]. They have

For peer review only - http://bmjopen.bmj.com/site/about/guidelines.xhtml

primarily investigated the effectiveness of strategies for reducing unprofessional behaviours but do not always explain why they do or do not work.

Our realist review differs from these by focussing on unprofessional behaviours among all staff to produce knowledge to enable more detail regarding the causes of such behaviours as well as improve context-specific understanding of how, why and in what circumstances unprofessional behaviours between staff in acute healthcare settings can be best understood, mitigated, managed, and prevented. Lastly, we will look beyond single professions to consider multiple professional groups and associated team-based and organisational interprofessional dynamics, which may impact on how unprofessional behaviours emerge or are addressed.

Methods

Review aim, objectives, and research questions

Aim

This realist review aims to improve context-specific understanding of how, why and in what circumstances unprofessional behaviours between staff in acute healthcare settings occur, and how strategies can be implemented to mitigate, manage, and prevent them.

Objectives

This review seeks to:

1. Conceptualise and refine terminology: by mapping behaviours defined as unprofessional to understand differences and similarities between terms referring to unprofessional behaviours (e.g., incivility, bullying, micro-aggressions, etc.) and how these terms are used by different professional groups in acute healthcare settings
2. Develop and refine Context, Mechanism and Outcome configurations (CMOCs): to understand the causes and contexts of unprofessional behaviours; the mechanisms which trigger different behaviours; and the outcomes on staff, patients, and wider system of healthcare
3. Identify strategies designed to mitigate, manage, and prevent unprofessional behaviours and explore how, why and in what circumstances these work and whom they benefit.
4. Produce recommendations and comprehensive resources that support the tailoring, implementation, monitoring and evaluation of contextually-sensitive strategies to tackle unprofessional behaviours and their impacts.

Research questions

- How are unprofessional behaviours defined, developed, and experienced by staff in acute healthcare settings?
- How do current strategies to reduce unprofessional behaviours in acute care address these behaviours or not?

For peer review only - http://bmjopen.bmj.com/site/about/guidelines.xhtml

- What are the mechanisms acting at individual, group, professional, and organisational levels that underpin strategies aimed at reducing unprofessional behaviours?
- What are the outcomes of unprofessional behaviours on staff (well-being; psychological safety), organisations (recruitment, turnover) and patients (e.g., patient safety and care quality)?
- What are the contexts which determine whether the different mechanisms produce their intended outcomes?
- What changes are needed to existing and/or future strategies to make them more effective?

Study design

This study uses a realist review methodology. Realist reviews are theory-driven and synthesise literature about complex social interventions [38]. They focus on understanding the mechanisms by which strategies work (or not) and seek to understand contextual influences on whether, why, how and for whom these might work [39]. In a realist understanding, contexts are either 1) observable features (space, place, people, things) that trigger or block an intervention with activation on a continuum, rather than a binary trigger (on/off switch) [40] or 2) relational and dynamic features that shaped the mechanisms through which an intervention works [41]. Likewise, mechanisms are interpreted as changes to participant reasoning in response to resources (e.g., education about unprofessional behaviours) introduced by an intervention [40].

The realist approach to data collection and analysis is driven by retroduction, a form of logical inference, which starts with the empirical, and explains outcomes and events by identifying the underlying mechanisms which are capable of producing them [42]. It is therefore essential to consider not only the specific unprofessional behaviours within the healthcare workforce, but any differences and similarities between staff groups as well (e.g., by specialty, professional group, setting, or seniority). By illuminating these contexts and working practices, we will also be able to determine how they might influence the presence or minimisation of unprofessional behaviours between healthcare staff working in acute settings.

This review will follow the Realist and Meta-Review Evidence Synthesis: Evolving Standards (RAMESES) guidelines on quality and reporting [43]. An overview of the review process is shown in Figure 1, with the steps we will take to achieve our objectives explained in the following sections

For peer review only - http://bmjopen.bmj.com/site/about/guidelines.xhtml

BMJ Open

[Figure 1 Location]

Figure 1. Review flow diagram. Dotted line reflects elements that may potentially happen.

For peer review only

Public, patient, and stakeholder involvement

We have worked closely with a stakeholder group in formulation of the proposal and have built in engagement as a key component to inform theory development. These stakeholders come from a range of relevant backgrounds, including patients and the public, heads of professional standards bodies, members of regulatory bodies and unions in the UK, influential theorists in the field, healthcare professionals with lived experience of unprofessional behaviours, and minority ethnic perspectives. This protocol incorporates four timepoints where stakeholder feedback will be incorporated (figure 1). This includes sense-checking our initial programme theories, aiding in identifying the most relevant evidence, ensuring our CMOCs reflect lived experience, and refining the final programme theories. In line with the ACTIVE (**A**uthors and **C**onsumers **T**ogether **I**mpacting on e**V**idencE) framework for stakeholder involvement in systematic reviews, our methodology is analogous to a continuous, multiple-time closed event approach with stakeholders able to influence the results of the review [44].

Stakeholder group feedback will be incorporated using the following processes to ensure rigour and relevance: 1) we will record what and how aspects of the developing theory are presented to stakeholders for refinement; 2) we will record where any alterations are suggested; 3) where suggestions are not aligned with what has been identified in the literature, we will perform purposive searching to sense-check recommendations; 4) any remaining discrepancies will be discussed within the team to determine majority consensus and 5) re-presentation of changes made in response to feedback to individual stakeholders or the whole group to further sense-check any changes [45]. We will take detailed notes of the meetings with the aid of a project administrator and will follow up with stakeholders individually regarding specific points raised.

Step 1: Identifying existing theories

To develop initial programme theories, we will iteratively:

a) Draw on preliminary discussions within the project team, with the healthcare workforce, patients, and the public;
b) Consult with our multidisciplinary stakeholder group as outlined above;
c) Examine healthcare literature already known to the research team (namely papers, reviews and reports identified in our initial scoping review which informed the funding proposal development), informal searching of key organisational websites (e.g., King's Fund, BMA, NHS England) and use of the 26 behaviours reported by Westbrook et al. for comparison against behaviours identified in the literature [2].

This first informal screening of the literature will sensitise the team to the breadth and depth of published and unpublished literature on unprofessional behaviours within healthcare. By investigating the theoretical underpinnings of programmes, we shall map the conceptual and theoretical landscape of unprofessional behaviour causes, outcomes, and how any identified strategies and interventions are theorised to work in acute healthcare settings. Building these initial programme theories will require iterative discussions within the project team and with our stakeholders to make sense of and synthesise the different assumptions. Once the programme theories have been developed by the project team, they will be presented to the stakeholder group to integrate their feedback and to ensure practical applicability (figure 1).

For peer review only - http://bmjopen.bmj.com/site/about/guidelines.xhtml

Step 2: Searching for evidence

Realist reviews typically draw on a combination of systematic searches, more informal, purposive searches, citation tracking and reference scanning [39]. It is also common to perform more searches later in the process in an evolutionary manner to further explain certain areas which are found to be underexplored in the programme theory. Additionally, in realist reviews, what is considered evidence often differs from other reviews. For example, non-empirical studies and commentaries can be included if they illuminate the topic being studied or can otherwise contribute to the programme theory. In realist reviews, the purpose is not to necessarily to identify every item of literature that could relate to a phenomenon, but, rather, to develop and refine a comprehensive theory to explain how the intervention works, why, for whom, and in what circumstances.

Search strategy

We will take a systematic approach and conduct searches to identify literature with which to test and refine our initial theories. In this formal, primary search we will:

a) Identify studies addressing strategies to reduce unprofessional behaviours in acute healthcare settings with all healthcare staff. Search academic databases including Medline, Embase and CINAHL and sources of trade, policy, and grey literature including Agency for Healthcare Research and Quality's Patient Safety Network (PSNET) and Google Scholar. Examples of trade journals we will search include AACN Bold Voices, Nursing Times, ED Management, and Nursing Standard. Search strategies will comprise search terms, synonyms, and index terms for: Acute care AND Healthcare staff AND Unprofessional behaviours. Searches from existing similar reviews such as Iling et al. (2013) will be consulted to aid identifying relevant search terms [36]. See supplementary file 1 for an indicative example search strategy, which is not complete, as searches are likely to evolve as is typical in a realist synthesis [39].
b) Conduct a grey literature search for professional codes of conduct produced by the Royal Colleges and the literature on cases brought to the Nursing and Midwifery Council, Health Care and Professions Council, and General Medical Council for unprofessional behaviour; using databases such as Health Management Information Consortium database and websites including NHS Employers, NHS Health Education England and Google (limited to screening the first 200 results).

Search strategies will be peer-reviewed using the PRESS (Peer Review of Electronic Search Strategies) method [46]. All search results will be saved in reference management software. A detailed, shared spreadsheet will record all searches conducted, to ensure transparency when reporting the search activities.

Additional searching

If we find that we require more data to develop, confirm, refute, or refine programme theory development, we will conduct additional purposive searches using CLUSTER search methods [47] to further develop any areas of the theory which require greater evidence to fully understand. These and earlier searches will follow the iterative realist search methods described by Booth et al. (2020) [48].

For peer review only

For peer review only - http://bmjopen.bmj.com/site/about/guidelines.xhtml

Step 3: Article selection

Inclusion and exclusion criteria

We will include both peer-reviewed and grey literature which help understand how and why strategies to reduce unprofessional behaviours in acute care settings work and whom they benefit. The following inclusion criteria will be used (table 1).

Table 1. Inclusion criteria.

Category	Criterion
Study design	Any (including non-empirical papers/ reports).
Study setting	Acute healthcare settings - acute, critical, emergency (and potentially wider, see relevance criteria below).
Types of unprofessional behaviour	All as exhibited and experienced by healthcare staff (not patients nor patient to staff).
Types of participants	All employed staff groups including students on placements.
Types of interventions/strategies	Individual, team, organisational, and policy level interventions. Cyber-bullying and other forms of online unprofessional behaviour included if it is staff-to-staff only.
Causes of unprofessional behaviours	All.
Outcomes	Included but not limited to all sources that focused on one or more of the following aspects: staff wellbeing (stress, burnout, resilience) staff turnover, absenteeism, malpractice claims, patient complaints, magnet hospital/recruitment, patient safety (avoidable harm, errors, speaking up rates, safety incidents, improved listening/response), cost.
Language	Only sources in English will be included.

Searches will not be limited (e.g., by date or publication) but records tagged as ‘children’, ‘animals’ or ‘elder abuse’ will be removed, and we will seek to exclude papers describing staff to patient or patient to staff unprofessional behaviours.

Screening, relevancy, and rigour

Screening of search results will be primarily undertaken by JAA in collaboration with JM, MP or RA who will screen a subset of records retrieved. A 10% random sub-sample of the citations retrieved from searching will be reviewed independently for quality control (by a second reviewer, either JM, MP or RA) at title and abstract, full text, and relevancy stages. Any disagreements will be resolved by discussion between JA, the second reviewer and JM. If disagreements remain, then a third member of the team (AJ, RM or JMW) will review and any disagreements will be resolved through further review/discussion, with final decision

For peer review only - http://bmjopen.bmj.com/site/about/guidelines.xhtml

resting with JM. The remaining 90% of decisions at these stages will be made by JAA. Screening of titles and abstracts will be performed using Rayyan.ai software (http://www.rayyan.ai/) and full texts will be screened using Mendeley (Mendeley Ltd.) [49].

Beyond the inclusion and exclusion criteria above, decisions regarding the inclusion or exclusion of sources will be based on a combination of relevance (based on both the major/minor criteria below and the ability to inform programme theories, i.e. conceptual richness and depth of sources) and rigour (whether the methods used to generate the relevant data are credible and trustworthy). Assessment of rigour will focus on the extent to which sources provide a detailed description of their methods and how generalisable and trustworthy their findings are based on those methods [39].

Our formal criteria for classifying the potential relevancy of sources are, by ascending order of relevancy:

Major contribution:

- Sources which contribute to the study aims and are conducted in an NHS context in acute care; or,
- Sources which contribute to the study aims and are conducted in an NHS context; or,
- Sources which contribute to the study aims and are conducted in contexts with similarities to the NHS (e.g., universal, publicly-funded health-care systems);

Minor contribution:

- Sources conducted in non-UK health-care systems that are markedly different to the NHS (e.g., fee-for-service, private insurance scheme systems) but where the mechanisms causing or moderating unprofessional behaviours could plausibly operate in the context of those working in the NHS; or,
- Sources which contribute to the study aims and can clearly help to identify mechanisms which could plausibly operate in the context of the NHS (e.g., law, police, and army).

We will prioritise the papers of major relevance in relation to the above criteria. These sources will then be sorted into the above categories in a reference manager and assessed for their ability to inform the refinement of programme theories (theoretical relevancy and conceptual richness). If we are unable to develop and refine aspects of the programme theory due to scarcity in majorly relevant sources, we will then draw on literature from the minor relevancy criteria above.

Step 4: Data extraction

In keeping with a realist retroductive analysis, relevant sections of texts that have been interpreted as related to contexts, mechanisms and/or their relationships to outcomes will be coded and organised in NVivo 12 software (QSR International) . This coding will be both inductive (codes created to categorise data reported in included sources) and deductive (codes created in advance of data extraction and analysis as informed by the initial programme theory). Each new element of relevant data will be used to test and refine aspects of the programme theory, and as it is refined, included sources will be re-scrutinised to search for data relevant to the revised programme theory that may have been missed

For peer review only - http://bmjopen.bmj.com/site/about/guidelines.xhtml

initially. The characteristics of the included sources will be extracted separately into an Excel spreadsheet to provide a descriptive overview of (but not limited to) settings, intervention, participants, and context.

As outlined above, we will start the coding and analysis process using the literature that has been deemed to make a 'major' i.e., most relevant contribution to the research questions to start building and refining our programme theory, while progressively focusing the review. Articles categorised as providing 'minor' contributions will be held back and analysed if there is no 'major' study which sheds light on certain aspects of the programme theory. The aim of the review will be to reach theoretical saturation in relation to the objectives, rather than to aggregate every single study that exists in the area. All study-related decisions will be documented and recorded as part of an audit trail to increase transparency and ensure consistency and rigour.

Step 5: Synthesising evidence and drawing conclusions

Our data analysis will use realist logic to make sense of the initial programme theory. Data will be interrogated at individual, team, and organisational levels to establish their relationships. This type of analysis will enable us to understand how the most relevant and important mechanisms work in different contexts, thus, allowing us to build more transferable context-mechanism-outcome configurations – these will be fully formulated by the end of this step. During the review, we will move iteratively between the analysis of particular examples from the literature, refinement of programme theory, and further iterative searching for data to test particular subsections of the programme theory as required (figure 1). We will also use the following strategies to make sense of the data (table 2) [50,51].

Table 2. Strategies foundational to the realist analysis.

**Strategies for synthesising evidence**
Compare and contrast sources of evidence – for example, where evidence about interventions or its mechanisms in one source allows insights into evidence about outcomes in another paper.
Reconciling of sources of evidence – where results differ in apparently similar circumstances, further investigation is appropriate in order to find explanations for why these different results occurred.
Adjudication of sources of evidence – this will enable us to make the synthesis more manageable. This includes strategies outlined in 'article selection' section above, i.e. dividing papers that make 'major' or 'minor' contributions to our research questions [51].
Consolidation of sources of evidence – where outcomes differ in particular contexts, an explanation can be constructed of how and why these outcomes occur differently.

Many of these processes are analogous to the identification of demi-regularities (*"semi-predictable patterns or pathways of programme functioning"* across studies). This type of thinking will be essential to help identify underlying mechanisms that are common to different strategies [40,52]. As required, we will also identify and incorporate middle range theories (e.g., theories around, for example, groupthink and psychological safety) to enable

For peer review only - http://bmjopen.bmj.com/site/about/guidelines.xhtml

us to move beyond description and understand the 'set of assumptions' underlying the observed associations [53].

Step 6: Testing and refining, and developing resources for stakeholders

We will then test and refine our programme theories with our stakeholder group. Informed by the 'Evidence Integration Triangle' [54] and stakeholder involvement at this final timepoint, we will use findings from our realist review to produce actionable evidence to support NHS managers/leaders to better understand how work environments may help or hinder unprofessional behaviours and identify what strategies work where.

Ethics and dissemination

Dissemination

Dissemination activities comprise step 7 of our project. The project will produce six major types of output in collaboration with our stakeholder group and will present some of these at our end of study project dissemination workshop (table 3).

Table 3. Dissemination and pathways to impact and who is reached [55].

Output	Description	Timescale to benefit	How will impact be achieved?	Who is reached?
**PPI / Stakeholder Engagement.**	PPI and our stakeholder representatives will be actively involved in the production of all outputs. The stakeholder group, including healthcare staff and PPI representatives, will be encouraged to think about alternative or additional approaches to dissemination.	During project and up to 6 months after.	Engaging with the stakeholder group will enable us to understand how best to reach people like them in the most well-targeted manner.	Internal stakeholder group of study and their immediate contacts.
**Media engagement strategy.**	We will identify the most appropriate way to engage with our non-Academic stakeholder groups. For example, through engagement with relevant professional bodies (e.g., British Medical Association, General Medical Council, Royal College of Nursing, Royal College of Midwives, NHS Employers etc) and through promoting our findings via alternative publication routes (e.g., Health Services Journal, Nursing Times/Standard,	During project and up to 2 years after.	Engaging with media will allow lighter touch but wider dissemination of our key messages.	Public, academics, practitioners, managers, leaders, policymakers.

For peer review only - http://bmjopen.bmj.com/site/about/guidelines.xhtml

BMJ Open

	BMJ, The Conversation, Twitter).			
**Resources for NHS managers/leaders.**	Tailored resources for NHS managers/leaders, and organisations supporting the healthcare workforce regarding how to implement strategies to reduce unprofessional behaviours.	End of project onwards.	Effects will be visible once stakeholders are able to implement changes and evaluate the impact of those changes.	Managers, leaders.
**Plain English summaries.**	We will create plain English summaries tailored to different audiences.	End of project and up to 2 years thereafter.	This will achieve impact through knowledge transfer in the short- to medium-term (1 month-2 years).	Healthcare professionals, managers, leaders, training providers, policymakers.
**Innovative forms of communication.**	We have had positive experiences of involving film makers and using the medium of theatre to perform research findings [56].	1-5 years.	Such outputs will create a longer-lasting impression with stakeholders than other more traditional forms of dissemination.	Healthcare professionals, managers, leaders, training providers, policymakers.
**Academic outputs.**	In addition to the main project report, an overall findings paper will be submitted to a high-impact peer-reviewed journal, and conference presentations at healthcare staff well-being conferences (such as Health Services Research UK) will be pursued.	3-5 years.	Informing the agenda for debate and action in health services and in public policy more widely.	Academics, managers, policymakers.

Ethical approval

The University of Surrey's Ethics committee have confirmed that ethical review is not needed for this realist review of secondary evidence.

Author contributions

JM led the conceptualisation and development of the overall study design and drafting of the protocol on which this paper is based. JAA drafted the manuscript in dialogue with JM and all authors and helped to refine the communication of the study methodology. RA, MP, JMW, AJ, RM and JW all contributed to the study conceptualisation and design of the study protocol. All authors substantively revised drafts of this manuscript and approved the final version.

For peer review only - http://bmjopen.bmj.com/site/about/guidelines.xhtml

Funding statement

This project was supported by the NIHR HS&DR Programme with grant number 131606. The views and opinions expressed herein are those of the authors and do not necessarily reflect those of the HS&DR programme.

Competing interests statement

None declared.

Patient consent

Not required.

References

1 Katz D, Blasius K, Isaak R, *et al.* Exposure to incivility hinders clinical performance in a simulated operative crisis. *BMJ Qual Saf* 2019;**28**:750–7. doi:10.1136/bmjqs-2019-009598

2 Westbrook J, Sunderland N, Li L, *et al.* The prevalence and impact of unprofessional behaviour among hospital workers: a survey in seven Australian hospitals. *Med J Aust* 2021;**214**:31–7. doi:10.5694/mja2.50849

3 Carter M, Thompson N, Crampton P, *et al.* Workplace bullying in the UK NHS: A questionnaire and interview study on prevalence, impact and barriers to reporting. *BMJ Open* 2013;**3**. doi:10.1136/bmjopen-2013-002628

4 Jones A, Blake J, Banks C, *et al.* Speaking up about Bullying and Harassment in Healthcare: Reflections Following the Introduction of an Innovative "Speak Up" Role in NHS England. In: *Connecting Healthcare Worker Well-Being, Patient Safety and Organisational Change*. Springer 2020. 145–61.

5 Felblinger DM. Bullying, incivility, and disruptive behaviors in the healthcare setting: identification, impact, and intervention. *Front Health Serv Manage* 2009;**25**:13–23. doi:10.1097/01974520-200904000-00003

6 Cruz D, Rodriguez Y, Mastropaolo C. Perceived microaggressions in health care: A measurement study. *PLoS One* 2019;**14**:1–11. doi:10.1371/journal.pone.0211620

7 Westbrook J, Sunderland N, Atkinson V, *et al.* Endemic unprofessional behaviour in health care: the mandate for a change in approach. *Med J Aust* 2018;**209**:380–1. doi:10.5694/mja17.01261

8 NHS England. NHS staff survey 2020. 2021. https://www.nhsstaffsurveys.com/

9 NHS Equality and Diversity Council. WRES 2018. Published Online First: 2018.https://www.england.nhs.uk/wp-content/uploads/2018/12/wres-2018-report-v1.pdf

10 Sue DW. *Microaggressions in everyday life: Race, gender, and sexual orientation*. John Wiley & Sons 2010.

11 Wild JRL, Ferguson HJM, McDermott FD, *et al.* Undermining and bullying in surgical training: A review and recommendations by the Association of Surgeons in Training. *Int J Surg* 2015;**23**:S5–9. doi:10.1016/j.ijsu.2015.09.017

For peer review only - http://bmjopen.bmj.com/site/about/guidelines.xhtml

BMJ Open

12 Pisklakov S, Tilak V, Patel A, *et al.* Bullying and Aggressive Behavior among Health Care Providers: Literature Review. *Adv Anthropol* 2013;**03**:179–82. doi:10.4236/aa.2013.34024

13 Ariza-Montes A, Muniz NM, Montero-Simó MJ, *et al.* Workplace bullying among healthcare workers. *Int J Environ Res Public Health* 2013;**10**:3121–39. doi:10.3390/ijerph10083121

14 Kline R, Lewis D. The price of fear: Estimating the financial cost of bullying and harassment to the NHS in England. *Public Money Manag* 2019;**39**:166–74. doi:10.1080/09540962.2018.1535044

15 Mannion R, Davies H. Raising and responding to frontline concerns in healthcare. *BMJ* 2019;**366**:1–4. doi:10.1136/bmj.l4944

16 Rosenstein AH. Nurse-physician relationships: Impact on nurse satisfaction and retention. *Am J Nurs* 2002;**102**:26–34. doi:10.1097/00000446-200206000-00040

17 NHS Employers. Tackling bullying & harassment in the NHS. Tackling bullying NHS infographic. 2019.https://www.nhsemployers.org/articles/tackling-bullying-nhs-infographic

18 Lever I, Dyball D, Greenberg N, *et al.* Health consequences of bullying in the healthcare workplace: A systematic review. *J Adv Nurs* 2019;**75**:3195–209. doi:10.1111/jan.13986

19 Nielsen MB, Indregard A-MR, Overland S. Workplace bullying and sickness absence: a systematic review and meta-analysis of the research literature. 2016.

20 Edmondson A. Psychological safety and learning behavior in work teams. 1999;**44**:350–83.https://doi.org/10.2307/2666999

21 Edmondson A. The Role of Psychological Safety. 2019. doi:10.1002/ltl.20419

22 Martinez W, Lehmann LS, Thomas EJ, *et al.* Speaking up about traditional and professionalism-related patient safety threats: A national survey of interns and residents. *BMJ Qual Saf* 2017;**26**:869–80. doi:10.1136/bmjqs-2016-006284

23 Barzallo Salazar MJ, Minkoff H, Bayya J, *et al.* Influence of surgeon behavior on trainee willingness to speak up: A randomized controlled trial. *J Am Coll Surg* 2014;**219**:1001–7. doi:10.1016/j.jamcollsurg.2014.07.933

24 Riskin A, Erez A, Foulk TA, *et al.* Rudeness and medical team performance. *Pediatrics* 2017;**139**. doi:10.1542/peds.2016-2305

25 Riskin A, Erez A, Foulk TA, *et al.* The impact of rudeness on medical team performance: A randomized trial. *Pediatrics* 2015;**136**:487–95. doi:10.1542/peds.2015-1385

26 Cooper WO, Spain DA, Guillamondegui O, *et al.* Association of Coworker Reports about Unprofessional Behavior by Surgeons with Surgical Complications in Their Patients. *JAMA Surg* 2019;**154**:828–34. doi:10.1001/jamasurg.2019.1738

27 Schoville R, Aebersold M. How Workplace Bullying and Incivility Impacts Patient Safety: A Qualitative Simulation Study Using BSN Students. *Clin Simul Nurs* 2020;**45**:16–23. doi:10.1016/j.ecns.2020.01.003

28 Doran N, Fox F, Rodham K, *et al.* Lost to the NHS: A mixed methods study of why GPS leave practice early in England. *Br J Gen Pract* 2016;**66**:e128–34. doi:10.3399/bjgp16X683425

29 Hogh A, Mikkelsen E, Hansen Å. Individual Consequences of Workplace Bullying/Mobbing. In: *Bullying and Harassment in the Workplace*. 2010. 107–28. doi:10.1201/ebk1439804896-7

For peer review only - http://bmjopen.bmj.com/site/about/guidelines.xhtml

30 Woodrow C, Guest D. A comparison of factors associated with workplace bullying in three UK Healthcare Organisations. In: *New Challenges for a Healthy Workplace in Human Services*. Rainer Hampp Verlag 2011. 87–104.

31 British Medical Association. Workplace bullying and harassment of doctors A review of recent research. 2017. file:///Users/VWC/Downloads/Bullying and harassment research review v7 WEB.pdf

32 Armstrong N. Management of Nursing Workplace Incivility in the Health Care Settings: A Systematic Review. *Work Heal Saf* 2018;**66**:403–10. doi:10.1177/2165079918771106

33 Gillen PA, Sinclair M, Kernohan WG, *et al.* Interventions for prevention of bullying in the workplace (Review). *Cochrane Database Syst Rev* 2017;:12. doi:10.1002/14651858.CD009778.pub2

34 Mannion R, Davies H, Powell M, *et al.* Healthcare scandals and the failings of doctors: Do official inquiries hold the profession to account? *J Health Organ Manag* 2019;**33**:221–40. doi:10.1108/JHOM-04-2018-0126

35 Keller S, Yule S, Zagarese V, *et al.* Predictors and triggers of incivility within healthcare teams: A systematic review of the literature. *BMJ Open* 2020;**10**:1–15. doi:10.1136/bmjopen-2019-035471

36 Illing J, Carter M, Thompson NJ, *et al.* Evidence synthesis on the occurrence, causes , management of bullying and harassing behaviours to inform decision making in the NHS. 2013;**44**:54–168.

37 Raine G, Thomas S, Rodgers M, *et al. Workplace-based interventions to promote healthy lifestyles in the NHS workforce: a rapid scoping and evidence map*. 2020. doi:10.3310/hsdr08180

38 Pawson R, Greenhalgh T, Harvey G, *et al.* Realist review - A new method of systematic review designed for complex policy interventions. *J Heal Serv Res Policy* 2005;**10**:21–34. doi:10.1258/1355819054308530

39 Wong G, Westhorp G, Pawson R, *et al.* Realist Synthesis: RAMESES Training Materials. *RAMESES Proj* 2013;:55.

40 Dalkin SM, Greenhalgh J, Jones D, *et al.* What's in a mechanism? Development of a key concept in realist evaluation. 2015;**10**:49.https://doi.org/10.1186/s13012-015-0237-x

41 Greenhalgh J, Manzano A. Understanding 'context' in realist evaluation and synthesis. *Int J Soc Res Methodol* 2021;**00**:1–13. doi:10.1080/13645579.2021.1918484

42 Sayer RA. *Method in social science: A realist approach*. Psychology Press 1992.

43 Wong G, Greenhalgh T, Westhorp G, *et al.* RAMESES publication standards: realist syntheses. *BMC Med* 2013;**11**:1–14.

44 Pollock A, Campbell P, Struthers C, *et al.* Development of the ACTIVE framework to describe stakeholder involvement in systematic reviews. *J Heal Serv Res Policy* 2019;**24**:245–55. doi:10.1177/1355819619841647

45 Abrams R, Park S, Wong G, *et al.* Lost in reviews: Looking for the involvement of stakeholders, patients, public and other non-researcher contributors in realist reviews. *Res Synth Methods* 2021;**12**:239–47. doi:10.1002/jrsm.1459

46 McGowan J, Sampson M, Salzwedel DM, *et al.* PRESS Peer Review of Electronic Search Strategies: 2015 Guideline Statement. *J Clin Epidemiol* 2016;**75**:40–6. doi:10.1016/j.jclinepi.2016.01.021

47 Booth A, Harris J, Croot E, *et al.* Towards a methodology for cluster searching to

For peer review only - http://bmjopen.bmj.com/site/about/guidelines.xhtml

BMJ Open

provide conceptual and contextual "richness" for systematic reviews of complex interventions: case study (CLUSTER). *BMC Med Res Methodol* 2013;**13**:1–14.
48 Booth A, Briscoe S, Wright JM. The "realist search": A systematic scoping review of current practice and reporting. *Res Synth Methods* 2020;**11**:14–35.
49 Ouzzani M, Hammady H, Fedorowicz Z, *et al.* Rayyan-a web and mobile app for systematic reviews. *Syst Rev* 2016;**5**:1–10. doi:10.1186/s13643-016-0384-4
50 Pawson R. *Evidence-based policy: a realist perspective*. sage 2006.
51 Pearson M, Chilton R, Wyatt K, *et al.* Implementing health promotion programmes in schools: A realist systematic review of research and experience in the United Kingdom. *Implement Sci* 2015;**10**:1–20. doi:10.1186/s13012-015-0338-6
52 Gilmore B, McAuliffe E, Power J, *et al.* Data Analysis and Synthesis Within a Realist Evaluation: Toward More Transparent Methodological Approaches. *Int J Qual Methods* 2019;**18**:1–11. doi:10.1177/1609406919859754
53 Merton RK. On theoretical sociology: five essays, old and new. 1967.
54 Glasgow RE, Green LW, Taylor M V., *et al.* An evidence integration triangle for aligning science with policy and practice. *Am J Prev Med* 2012;**42**:646–54. doi:10.1016/j.amepre.2012.02.016
55 Lamont T. *Making Research Matter: Steps to Impact for Health and Care Researchers*. Policy Press 2021.
56 Maben J, Taylor C, Dawson J, *et al.* A realist informed mixed-methods evaluation of Schwartz Center Rounds® in England. *Heal Serv Deliv Res* 2018;**6**:1–260. doi:10.3310/hsdr06370

For peer review only

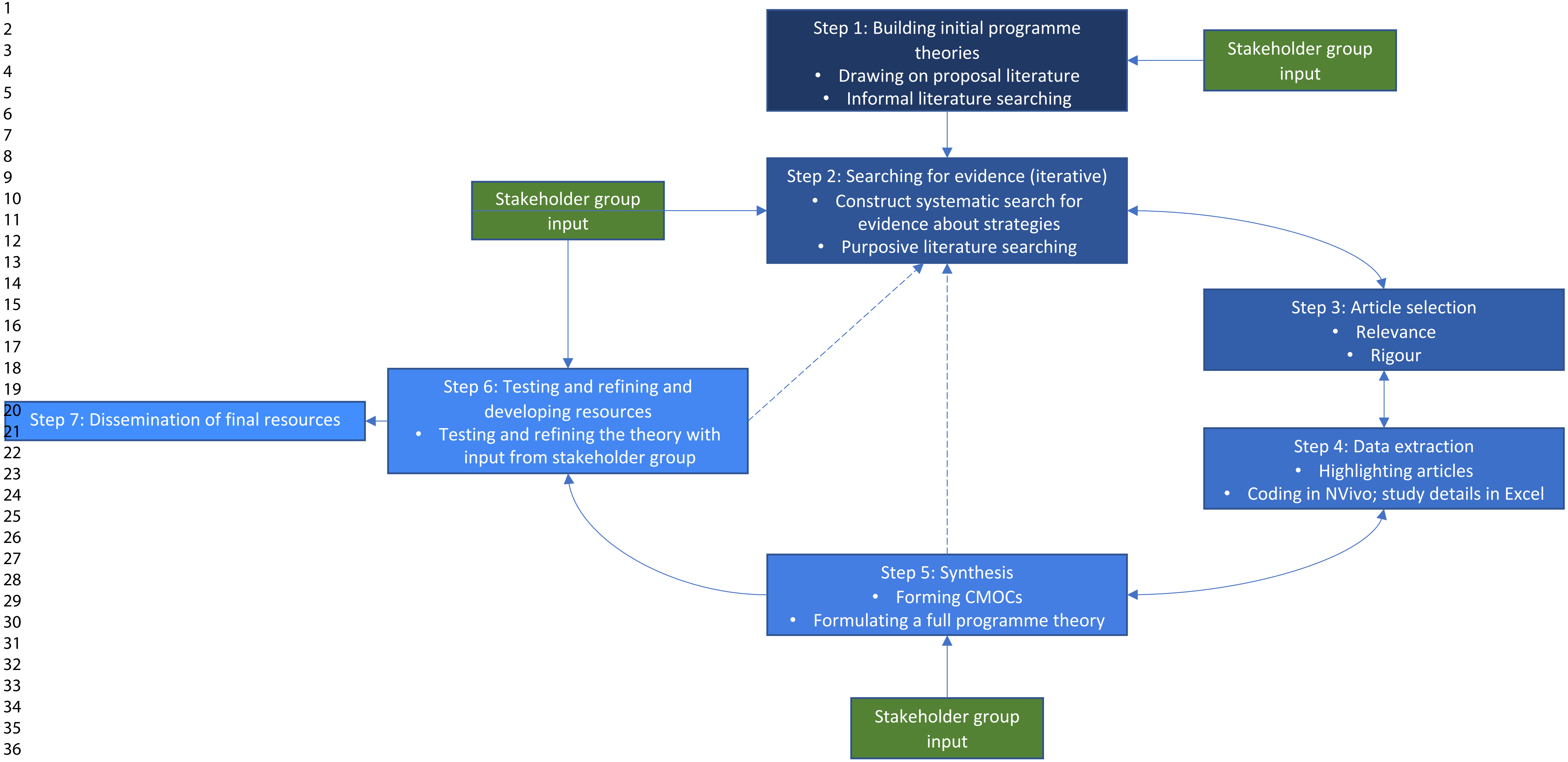
Step 1: Building initial programme theories
• Drawing on proposal literature
• Informal literature searching
Stakeholder group input
Step 2: Searching for evidence (iterative)
• Construct systematic search for evidence about strategies
• Purposive literature searching
Stakeholder group input
Step 3: Article selection
• Relevance
• Rigour
Step 4: Data extraction
• Highlighting articles
• Coding in NVivo; study details in Excel
Step 5: Synthesis
• Forming CMOCs
• Formulating a full programme theory
Stakeholder group input
Step 6: Testing and refining and developing resources
• Testing and refining the theory with input from stakeholder group
Step 7: Dissemination of final resources

Literature search strategy examples

Unprofessional Behaviours among Acute Care staff – MEDLINE systematic strategy (draft)

1. Ovid MEDLINE(R) ALL <1946 to February 01, 2022>

1 emergency medical services/ or advanced trauma life support care/ or call centers/ or emergency medical dispatch/ or emergency medical service communication systems/ 47376

2 exp airway management/ or exp emergency treatment/ or exp ambulatory care/ or exp critical care/ or exp perioperative care/ or exp preoperative care/ 456732

3 exp emergency service, hospital/ or emergency services, psychiatric/ or hotlines/ or poison control centers/ or exp "transportation of patients"/ or triage/ or critical care nursing/ or emergency nursing/ 129117

4 (emergenc* adj5 (care or service* or health* or ill* or treat* or medic* or unit* or centre* or centre* or department* or setting*)).tw,kf. 200510

5 (acute* adj5 (care or service* or health* or ill* or treat* or medic* or unit* or centre* or centre* or department* or setting* or ward?)).tw,kf. 220374

6 (trauma* adj5 (care or service* or ill* or unit* or centre* or centre* or department*)).tw,kf. 26629

7 (ambula* adj5 (care or service* or unit* or centre* or centre* or department* or setting*)).tw,kf. 26105

8 (critical* adj2 (care or ill*)).tw,kf. 92228

9 (urgent adj3 (care or service* or medic*)).tw,kf. 5298

10 "intensive care".tw,kf. 171169

11 or/1-10 [Acute Care or Ambulance services] 1058529

12 agonistic behavior/ 1778

13 exp bullying/ 5615

14 problem behavior/ 3232

15 exp harassment, non-sexual/ 5697

16 sexual harassment/ 2020

17 incivility/ 232

18 Professional Misconduct/ 3432

19 Hostility/ 5396

20 Social Discrimination/ 1494

21 bully*.tw,kf. 7035

22 harass*.tw,kf. 4646

For peer review only

23 intimidat*.tw,kf. 1506

24 (lateral* adj2 violence).tw,kf. 100

25 (horizontal* adj2 violence).tw,kf. 146

26 (transgressive adj3 behavio?r*).tw,kf. 39

27 (disruptive adj3 behavio?r*).tw,kf. 4362

28 (unprofessional adj3 behavio?r*).tw,kf. 399

29 (micro-aggress* or microaggress*).tw,kf. 474

30 incivil*.tw,kf. 683

31 uncivil*.tw,kf. 212

32 rude*.tw,kf. 1193

33 mistreat*.tw,kf. 2441

34 (professional* adj3 misconduct).tw,kf. 303

35 mobbing.tw,kf. 416

36 (negative behavio* or negative act?).tw,kf. 1713

37 or/12-36 [Unprofessional behaviours] 40104

38 exp Health Personnel/ 571903

39 exp Students, Health Occupations/ 79772

40 exp education, graduate/ or "internship and residency"/ or teaching rounds/ 94345

41 exp Interprofessional Relations/ 71796

42 Workplace/ 26778

43 (nurs* or midwif* or midwiv*).tw,kf. 516310

44 paramedic?.tw,kf. 6034

45 (doctor? or physician? or clinician? or surgeon? or consultant?).tw,kf. 1002988

46 (student? adj2 (medic* or health* or clinic*)).tw,kf. 65732

47 intern?.tw,kf. 11160

48 resident?.tw,kf. 178976

49 (Therapist? or Pharmacist? or Optometrist? or Nutritionist? or Dentist? or Physiotherapist?).tw,kf. 132472

50 (Audiologist? or Anatomist? or Allergist? or An?esthetist? or An?esthesiologist? or Cardiologist? or Endocrinologist? or Gastroenterologist? or GP? or Geriatrician? or Hospitalist? or Oncologist?).tw,kf. 225380

For peer review only

51 (Ophthalmologist? or Otolaryngologist? or Pathologist? or P?ediatrician? or Physiatrist? or Psychiatrist? or Pulmonologist? or Radiographer? or Radiologist?).tw,kf. 170433

52 medic?.tw,kf. 21462

53 assistant?.tw,kf. 28781

54 (cleaner? or ancillary or porter?).tw,kf. 24901

55 (auxillary or auxillaries or administrator? or secretary or secretaries or receptionist? or technician?).tw,kf. 41498

56 (employee? or worker? or Staff or personnel or practitioner? or professional? or workforce*).tw,kf. 917727

57 (workplace* or "work place*" or worksite* or "work site*" or "work setting*").tw,kf. 55952

58 or/38-57 [Staff] 2959526

59 37 and 58 [UB and Staff search 1] 14614

60 physician-nurse relations/ or interprofessional relations/ 54899

61 aggression/ or prejudice/ or ageism/ or gender equity/ or homophobia/ or racism/ or sexism/ or weight prejudice/ or xenophobia/ 69260

62 60 and 61 [Aggression or prejudice among staff MeSH] 740

63 ((staff or employee* or work* or nurs* or doctor?) adj8 (WPV or violen*) adj5 among*).tw,kf. 560

64 ((staff or employee* or work* or nurs* or doctor?) adj8 Victim* adj5 among*).tw,kf. 109

65 ((staff or employee* or work* or nurs* or doctor?) adj8 hostil*).tw,kf. 497

66 ((staff or employee* or work* or nurs* or doctor?) adj3 undermin*).tw,kf. 334

67 ((staff or employee* or work* or nurs* or doctor?) adj6 discriminat*).tw,kf. 3201

68 "abusive supervision".tw,kf. 140

69 (workplace adj3 conflict*).tw,kf.174

70 ((staff or employee* or work* or nurs* or doctor?) adj5 (gender adj2 (inequalit* or equalit* or discriminat*))).tw,kf. 281

71 ((staff or employee* or work* or nurs* or doctor?) adj5 (racism or racist or (racial adj3 abus*))).tw,kf. 330

72 ((staff or employee* or work* or nurs* or doctor?) adj5 (sexism or sexist)).tw,kf. 83

73 ((staff or employee* or work* or nurs* or doctor?) adj8 ((disabilit* or disabled) adj5 (inequalit* or equalit* or discriminat*))).tw,kf. 97

74 ((staff or employee* or work* or nurs* or doctor?) adj8 ableis*).tw,kf. 6

75 or/62-74 [UB and staff search 2]6234

For peer review only

76 59 or 75 [UB among Staff final search] 20009

77 11 and 76 [UB among Staff in Acute Care] 960

78 afghanistan/ or africa/ or africa, northern/ or africa, central/ or africa, eastern/ or "africa south of the sahara"/ or africa, southern/ or africa, western/ or albania/ or algeria/ or andorra/ or angola/ or "antigua and barbuda"/ or argentina/ or armenia/ or azerbaijan/ or bahamas/ or bahrain/ or bangladesh/ or barbados/ or belize/ or benin/ or bhutan/ or bolivia/ or borneo/ or "bosnia and herzegovina"/ or botswana/ or brazil/ or brunei/ or bulgaria/ or burkina faso/ or burundi/ or cabo verde/ or cambodia/ or cameroon/ or central african republic/ or chad/ or exp china/ or comoros/ or congo/ or cote d'ivoire/ or croatia/ or cuba/ or "democratic republic of the congo"/ or cyprus/ or djibouti/ or dominica/ or dominican republic/ or ecuador/ or egypt/ or el salvador/ or equatorial guinea/ or eritrea/ or eswatini/ or ethiopia/ or fiji/ or gabon/ or gambia/ or "georgia (republic)"/ or ghana/ or grenada/ or guatemala/ or guinea/ or guinea-bissau/ or guyana/ or haiti/ or honduras/ or independent state of samoa/ or exp india/ or indian ocean islands/ or indochina/ or indonesia/ or iran/ or iraq/ or jamaica/ or jordan/ or kazakhstan/ or kenya/ or kosovo/ or kuwait/ or kyrgyzstan/ or laos/ or lebanon/ or liechtenstein/ or lesotho/ or liberia/ or libya/ or madagascar/ or malaysia/ or malawi/ or mali/ or malta/ or mauritania/ or mauritius/ or mekong valley/ or melanesia/ or micronesia/ or monaco/ or mongolia/ or montenegro/ or morocco/ or mozambique/ or myanmar/ or namibia/ or nepal/ or nicaragua/ or niger/ or nigeria/ or oman/ or pakistan/ or palau/ or exp panama/ or papua new guinea/ or paraguay/ or peru/ or philippines/ or qatar/ or "republic of belarus"/ or "republic of north macedonia"/ or romania/ or exp russia/ or rwanda/ or "saint kitts and nevis"/ or saint lucia/ or "saint vincent and the grenadines"/ or "sao tome and principe"/ or saudi arabia/ or serbia/ or sierra leone/ or senegal/ or seychelles/ or singapore/ or somalia/ or south africa/ or south sudan/ or sri lanka/ or sudan/ or suriname/ or syria/ or taiwan/ or tajikistan/ or tanzania/ or thailand/ or timor-leste/ or togo/ or tonga/ or "trinidad and tobago"/ or tunisia/ or turkmenistan/ or uganda/ or ukraine/ or united arab emirates/ or uruguay/ or uzbekistan/ or vanuatu/ or venezuela/ or vietnam/ or west indies/ or yemen/ or zambia/ or zimbabwe/ 1196785

79 "Organisation for Economic Co-Operation and Development"/ 413

80 australasia/ or exp australia/ or austria/ or baltic states/ or belgium/ or exp canada/ or chile/ or colombia/ or costa rica/ or czech republic/ or exp denmark/ or estonia/ or europe/ or finland/ or exp france/ or exp germany/ or greece/ or hungary/ or iceland/ or ireland/ or israel/ or exp italy/ or exp japan/ or korea/ or latvia/ or lithuania/ or luxembourg/ or mexico/ or netherlands/ or new zealand/ or north america/ or exp norway/ or poland/ or portugal/ or exp "republic of korea"/ or "scandinavian and nordic countries"/ or slovakia/ or slovenia/ or spain/ or sweden/ or switzerland/ or turkey/ or exp united kingdom/ or exp united states/ 3378869

81 European Union/ 17082

82 Developed Countries/ 21055

83 or/79-82 3394093

84 78 not 83 [OECD search filter NICE 2021] 1110107

85 77 not 84 [UB among Acute Care Staff with non-OECD countries removed] 872

86 (exp Child/ or Adolescent/ or exp Infant/) not exp Adult/2018609

87 85 not 86 [Child studies removed] 804

For peer review only

88	(elder mistreat* or elder abuse* or elder neglect*).tw,kf.	1880
89	87 not 88 [Elder abuse studies removed]	780

Google Scholar search strategy examples

Google Scholar - Theories
Search 1
bullying | harassment | discrimination|unprofessional AND workplace|worker|staff AND model|framework|concept|idea|opinion|theory|view|perception|attitude|theories [Searched in Publish or Perish Title Field]

Search 2 – Interventions & strategies
interventions|strategies|techniques|program|programs|programme|programmes AND unprofessional|bullying|harassment|discrimination AND nurse|doctor|paramedic|hospital|ambulance|staff|professional AND emergency|acute|trauma [searched in Publish or Perish Keywords field]

For peer review only

	Reporting item	Description of item	Reported on page(s)
**Title**			
1		In the title, identify the document as a realist synthesis or review	1
**Abstract**			
2		While acknowledging publication requirements and house style, abstracts should ideally contain brief details of: the study's background, review question or objectives; search strategy; methods of selection, appraisal, analysis and synthesis of sources; main results; and implications for practice	1-2
**Introduction**			
3	Rationale for review	Explain why the review is needed and what it is likely to contribute to existing understanding of the topic area	4-5
4	Objectives and focus of review	State the objective(s) of the review and/or the review question(s). Define and provide a rationale for the focus of the review	5-6
**Methods**			

	Reporting item	Description of item	Reported on page(s)
5	Changes in the review process	Any changes made to the review process that was initially planned should be briefly described and justified	Potential changes outlined on pg 10-12
6	Rationale for using realist synthesis	Explain why realist synthesis was considered the most appropriate method to use	4
7	Scoping the literature	Describe and justify the initial process of exploratory scoping of the literature	8
8	Searching processes	While considering specific requirements of the journal or other publication outlet, state and provide a rationale for how the iterative searching was done. Provide details on all of the sources accessed for information in the review. Where searching in electronic databases has taken place, the details should include, for example, name of database, search terms, dates of coverage and date last searched. If individuals familiar with the relevant literature and/or topic area were contacted, indicate how they were identified and selected	9-10
9	Selection and appraisal of documents	Explain how judgements were made about including and excluding data from documents, and justify these	10-11
10	Data extraction	Describe and explain which data or information were extracted from the included documents and justify this selection	11-12

For peer review only

	Reporting item	Description of item	Reported on page(s)
11	Analysis and synthesis processes	Describe the analysis and synthesis processes in detail. This section should include information on the constructs analysed and describe the analytic process	12-13
	**Results**		
12	Document flow diagram	Provide details on the number of documents assessed for eligibility and included in the review, with reasons for exclusion at each stage, as well as an indication of their source of origin (e.g. from searching databases, reference lists and so on). You may consider using the example templates (which are likely to need modification to suit the data) that are provided	7
13	Document characteristics	Provide information on the characteristics of the documents included in the review	n/a
14	Main findings	Present the key findings with a specific focus on theory building and testing	n/a
	**Discussion**		
15	Summary of findings	Summarise the main findings, taking into account the reviews objective(s), research question(s), focus and intended audience(s)	n/a

Reporting item		Description of item	Reported on page(s)
16	Strengths, limitations and future research directions	Discuss both the strengths of the review and its limitations. These should include (but need not be restricted to) (a) consideration of all the steps in the review process and (b) comment on the overall strength of evidence supporting the explanatory insights which emerged<br>The limitations identified may point to areas where further work is needed	2 & 14
17	Comparison with existing literature	Where applicable, compare and contrast the reviews findings with the existing literature (e.g. other reviews) on the same topic	2-5
18	Conclusion and recommendations	List the main implications of the findings and place these in the context of other relevant literature. If appropriate, offer recommendations for policy and practice	14
19	Funding	Provide details of funding source (if any) for the review, the role played by the funder (if any) and any conflicts of interests of the reviewers	15

For peer review only

BMJ Open

Why do acute healthcare staff engage in unprofessional behaviours towards each other and how can these behaviours be reduced? A realist review protocol

Journal:	*BMJ Open*
Manuscript ID	bmjopen-2022-061771.R2
Article Type:	Protocol
Date Submitted by the Author:	23-May-2022
Complete List of Authors:	Maben, Jill; University of Surrey, School of Health Sciences<br>Aunger, Justin; University of Surrey, School of Health Sciences<br>Abrams, Ruth; University of Surrey, School of Health Sciences, Faculty of Health and Medical Sciences<br>Pearson, Mark; Hull York Medical School, Wolfson Palliative Care Research Centre<br>Wright, Judy; University of Leeds<br>Westbrook, Johanna; Macquarie University<br>Mannion, Russell; University of Birmingham, Health Services Management Centre<br>Jones, Aled; Cardiff University
**Primary Subject Heading**:	Health services research
Secondary Subject Heading:	Health services research, Health policy, Nursing
Keywords:	Health & safety < HEALTH SERVICES ADMINISTRATION & MANAGEMENT, Health policy < HEALTH SERVICES ADMINISTRATION & MANAGEMENT, Quality in health care < HEALTH SERVICES ADMINISTRATION & MANAGEMENT, Organisation of health services < HEALTH SERVICES ADMINISTRATION & MANAGEMENT

SCHOLARONE™
Manuscripts

BMJ Open

Why do acute healthcare staff engage in unprofessional behaviours towards each other and how can these behaviours be reduced? A realist review protocol

Prof. Jill Maben[1,*], Dr. Justin Avery Aunger[1], Dr. Ruth Abrams[1], Dr. Mark Pearson[2], Ms. Judy M Wright[3], Prof. Johanna Westbrook[4], Prof. Russell Mannion[5], Prof. Aled Jones[6].

*Corresponding author

Affiliations:
[1] School of Health Sciences, Faculty of Health & Medical Sciences, University of Surrey, Kate Granger Building, Priestly Road, Surrey Research Park, Guildford, Surrey, GU2 7YH, United Kingdom
[2] Wolfson Palliative Care Research Centre, Hull York Medical School, Allam Medical Building, University of Hull, Hull, HU6 7RX, United Kingdom
[3] Leeds Institute of Health Sciences, Worsley Building, University of Leeds, Leeds, LS2 9NL, United Kingdom
[4] Centre for Health Systems and Safety Research, Australian Institute of Health Innovation, Macquarie University, Australia.
[5] Health Services Management Centre, Park House, University of Birmingham, Birmingham, B15 2RT, United Kingdom
[6] School of Healthcare Sciences, College of Biomedical and Life Sciences, Cardiff University, Eastgate House, 35-43 Newport Road, Cardiff, CF24 0AB, United Kingdom

Email addresses:
Jill Maben: J.Maben@surrey.ac.uk
Justin Avery Aunger: J.Aunger@surrey.ac.uk
Ruth Abrams: R.Abrams@surrey.ac.uk
Mark Pearson: Mark.Pearson@hyms.ac.uk
Judy M Wright: J.M.Wright@Leeds.ac.uk
Johanna Westbrook: johanna.westbrook@mq.edu.au
Russell Mannion: r.mannion@bham.ac.uk
Aled Jones: JonesA97@cardiff.ac.uk

Word count: 4139

Abstract

Introduction

Unprofessional behaviours encompass many behaviours including bullying, harassment, and microaggressions. These behaviours between healthcare staff are problematic; they affect people's ability to work, to feel psychologically safe at work and speak up, and to deliver safe care to patients. Almost a fifth of UK NHS staff experience unprofessional behaviours in the workplace, with higher incidence in acute care settings and for staff from minority backgrounds. Existing analyses have investigated the effectiveness of strategies at reducing these behaviours. We seek to go beyond these, to understand the range and causes of such behaviours, their negative effects, and how mitigation strategies may work, in which contexts, and for whom.

For peer review only - http://bmjopen.bmj.com/site/about/guidelines.xhtml

For peer review only

Methods and analysis

This study uses a realist review methodology with stakeholder input comprising a number of iterative steps: (1) formulating initial programme theories drawing on informal literature searches and literature already known to the study team, (2) performing systematic and purposive searches for grey and peer-reviewed literature on Embase, CINAHL, and Medline databases as well as Google and Google Scholar, (3) selecting appropriate documents while considering rigour and relevance, (4) extracting data, (5) and synthesising and (6) refining the programme theories by testing the theories against the newly identified literature.

Ethics and dissemination

Ethical review is not required as this study is secondary research. An impact strategy has been developed which includes working closely with key stakeholders throughout the project. Step 7 of our project will develop pragmatic resources for managers and professionals, tailoring contextually-sensitive strategies to reduce unprofessional behaviours, identifying what works for which groups. We will be guided by the 'Evidence Integration Triangle' to implement the best strategies to reduce unprofessional behaviours in given contexts. Dissemination will occur through presentation at conferences, innovative methods (cartoons, videos, animations and/or interactive performances), and peer-reviewed journals.

PROSPERO Registration:

This review is registered at PROSPERO with ID CRD42021255490.

Keywords:

Incivility; bullying; professionalism; psychological safety; workforce

Article summary

Strengths and Limitations

- This review is the first to identify the causes of a wide range of unprofessional behaviours (not only bullying and harassment) among staff for a range of staff groups and contexts in practice.
- The review utilises a context-sensitive realist methodology that accounts for complexity, to understand how and why strategies to reduce staff-to-staff unprofessional behaviours in acute care settings work and for whom.
- Reviewing both the causes of unprofessional behaviours and the strategies to reduce them will enable us to gain a better understanding of how strategies interact with and target the underlying mechanisms that lead to unprofessional behaviours.
- This review includes stakeholder engagement throughout, including refinement of programme theory and co-creation of actionable, context-specific outputs.
- Limitations of the review include a primary focus on acute healthcare settings, the inclusion of sources published only in English, and focus on health systems with similarities to a UK healthcare context.

For peer review only - http://bmjopen.bmj.com/site/about/guidelines.xhtml

For peer review only

Introduction

Unprofessional behaviours can negatively impact staff wellbeing, patient safety and have organisational cost implications [1,2]. They are a pervasive issue in workplaces throughout global healthcare systems [2–4]. The term unprofessional behaviours is a generic one which includes a range of more specific behaviours such as uncivil, transgressive or disruptive behaviours, physical and verbal aggression [5] and bullying [6]. Unprofessional behaviours have been defined as "*a wide spectrum that includes conduct that more subtly interferes with team functioning, such as poor or ambiguous communication, passive aggression, lack of responsiveness, public criticism of colleagues, and humour at others' expense*" [7]. As such, unprofessional behaviours can be casual and generalised, or highly targeted with the intention to cause harm. Unprofessional behaviours are increasingly being recognised as unacceptable in a modern, inclusive health setting, as they detrimentally affect the work and psychological wellbeing of others.

Prevalence of unprofessional behaviours in the UK healthcare system

Workplace bullying affects staff in all roles and at all levels throughout the UK healthcare system. The 2020 UK National Health Service (NHS) staff survey found that 18.7% of all staff reported experiencing bullying or harassment at work from their colleagues [8], and 12.4% of staff reported experiencing these behaviours from managers [8]. Staff in ambulance service trusts and certain minority groups experience above average rates of bullying and harassment [9], including women, individuals with a disability, and members of minority communities, through micro-aggressions [6,10] and harassment or discrimination [11–13]. For example, the 2020 staff survey showed that minority ethnic respondents reported 5.6% higher rates of bullying and harassment from other staff than did white respondents [8].

Impacts of unprofessional behaviours

Unprofessional behaviours can have negative impacts on staff psychological wellbeing in the workplace, on patient safety and quality of care, and for wider systems and culture [14–17]. For example, bullying of individual staff members in the healthcare workplace can lead to psychological issues such as depression, burnout, and distress [18]. Bullying can also lead to physical problems such as sleep disturbance, headache, and gastrointestinal upset, resulting in staff taking sick leave [2]. In extreme cases, bullying and harassment can lead to suicidal ideation [19].

Unprofessional behaviours can result in staff feeling unsafe at work and an absence of psychological safety may encourage a climate of unprofessional behaviours and inhibit speaking up [1]. Psychological safety at work is absent in teams where incivility, transgressive behaviours, and bullying and harassment are experienced [20]. This can result in reduced staff psychological well-being at work and reduced job satisfaction, engagement, and motivation [21].

The impact of unprofessional behaviours on speaking up and patient safety is well documented [1,22,23]. Where a culture of psychological safety is encouraged, employees are more likely to speak up about errors in patient care [23]. Psychologically safe teams share significant information and perform better together, improving patient safety [24–26]. For example, a recent simulation study conducted in the USA with nursing students found

For peer review only - http://bmjopen.bmj.com/site/about/guidelines.xhtml

BMJ Open

that where there was bullying the work environment became chaotic, overwhelming, and increased the prevalence of unsafe practices [27]. Another study performed in Israel found that team-targeted rudeness alone explained 12% of the variance in team care performance with significant negative clinical outcomes [25].

Unprofessional behaviours are associated with higher staff turnover and other significant financial costs such as litigation, lower staff productivity and sickness leave [28–31]. Kline & Lewis' (2019) conservative estimate of the financial cost of bullying and harassment to the NHS (due to sickness absence, employee turnover, reduced productivity, compensation, and litigation costs) suggests damages from bullying alone was at least £2.28 billion per annum, or 1.52% of the NHS' budget for 2019/2020 [14].

Strategies to reduce unprofessional behaviours

Unprofessional behaviours, such as bullying, constitute a complex problem that require a broad-ranging, strategic approach that incorporates multiple levels of analysis [7]. One systematic review of workplace incivility in nursing for example, identified many potential strategies [32]; these included education about workplace incivility and its impacts, assertiveness training, rehearsal of responding to incivility, and teambuilding exercises. There are also policy-level initiatives, such as legislation against harassment that is currently enshrined in UK and European law [33]. A recent Cochrane review [33] identifies that strategies can operate on society/policy, organisation, job/task, and individual/job interface levels. Such strategies can also be preventative, ameliorative, or reactive, in nature [33].

To date, much of the policy focus with regard to addressing unprofessional behaviours in healthcare settings has been on identifying 'bad apples' (individual instigators), with a more recent focus on the role of the organisational environments and cultures in which they work ('bad barrels') [34]. There is also a role for healthcare professions regulators to regulate their own organisations ('bad cellars'), as well as considering the shifting wider political and policy context ('bad orchards') [34]. Capturing these different levels of analysis and interventions is a complex task, requiring a methodology capable of understanding how strategies interact to produce particular outcomes in different contexts and for whom. A realist approach is one such methodology.

Existing syntheses and our contribution

Our realist review will complement and extend other recent reviews [35,36]. These include, for example, a systematic review which identifies the predictors and triggers of staff incivility within healthcare teams, including high workload, communication issues, lack of support, poor leadership, and being more junior in a team [35]. Similarly, a realist-informed review of bullying and harassment concluded that the involvement of management and wider organisational commitment were key to intervention success [36]. While this review focused solely on bullying and harassment [36], others have mapped the terrain without providing sufficient granular detail to differentiate between professional groups and contexts, or have included unprofessional behaviours towards patients [37]. They have primarily investigated the effectiveness of strategies for reducing unprofessional behaviours but do not always explain why they do or do not work.

For peer review only - http://bmjopen.bmj.com/site/about/guidelines.xhtml

Our realist review differs from these by focussing on unprofessional behaviours among all staff to produce more detailed knowledge regarding the causes of such behaviours as well as improve context-specific understanding of how, why and in what circumstances unprofessional behaviours between staff in acute healthcare settings occur, and how they can best be understood, mitigated, managed, and prevented. Lastly, we will look beyond single professions to consider multiple professional groups and associated team-based and organisational interprofessional dynamics, which may impact on how unprofessional behaviours emerge or are addressed.

Methods

Review aim, objectives, and research questions

Aim

This realist review aims to improve context-specific understanding of how, why and in what circumstances unprofessional behaviours between staff in acute healthcare settings occur, and how strategies can be implemented to mitigate, manage, and prevent them.

Objectives

This review seeks to:

1. Conceptualise and refine terminology: by mapping behaviours defined as unprofessional to understand differences and similarities between terms referring to unprofessional behaviours (e.g., incivility, bullying, micro-aggressions, etc.) and how these terms are used by different professional groups in acute healthcare settings
2. Develop and refine Context, Mechanism and Outcome configurations (CMOCs): to understand the causes and contexts of unprofessional behaviours; the mechanisms which trigger different behaviours; and the outcomes on staff, patients, and wider system of healthcare
3. Identify strategies designed to mitigate, manage, and prevent unprofessional behaviours and explore how, why and in what circumstances these work and whom they benefit.
4. Produce recommendations and comprehensive resources that support the tailoring, implementation, monitoring and evaluation of contextually-sensitive strategies to tackle unprofessional behaviours and their impacts.

Research questions

- How are unprofessional behaviours defined, developed, and experienced by staff in acute healthcare settings?
- How do current strategies to reduce unprofessional behaviours in acute care address these behaviours or not?
- What are the mechanisms acting at individual, group, professional, and organisational levels that underpin strategies aimed at reducing unprofessional behaviours?

For peer review only

BMJ Open

- What are the outcomes of unprofessional behaviours on staff (wellbeing, psychological safety), organisations (recruitment, turnover) and patients (e.g., patient safety and care quality)?
- What are the contexts which determine whether the different mechanisms produce their intended outcomes?
- What changes are needed to existing and/or future strategies to make them more effective?

Study design

This study uses a realist review methodology. Realist reviews are theory-driven and synthesise literature about complex social interventions [38]. They focus on understanding the mechanisms by which strategies work (or not) and seek to understand contextual influences on whether, why, how and for whom these might work [39]. In a realist understanding, contexts are either 1) observable features (space, place, people, things) that trigger or block an intervention with activation on a continuum, rather than a binary trigger (on/off switch) [40] or 2) relational and dynamic features that shaped the mechanisms through which an intervention works [41]. Likewise, mechanisms are interpreted as changes to participant reasoning in response to resources (e.g., education about unprofessional behaviours) introduced by an intervention [40].

The realist approach to data collection and analysis is driven by retroduction, a form of logical inference, which starts with the empirical, and explains outcomes and events by identifying the underlying mechanisms which are capable of producing them [42]. It is therefore essential to consider not only the specific unprofessional behaviours within the healthcare workforce, but any differences and similarities between staff groups as well (e.g., by specialty, professional group, setting, or seniority). By illuminating these contexts and working practices, we will also be able to determine how they might influence the presence or minimisation of unprofessional behaviours between healthcare staff working in acute settings.

This review will follow the Realist and Meta-Review Evidence Synthesis: Evolving Standards (RAMESES) guidelines on quality and reporting [43]. An overview of the review process is shown in Figure 1, with the steps we will take to achieve our objectives explained in the following sections

For peer review only - http://bmjopen.bmj.com/site/about/guidelines.xhtml

BMJ Open

[Figure 1 Location]

Figure 1. Review flow diagram. Dotted line reflects elements that may potentially happen.

For peer review only

For peer review only - http://bmjopen.bmj.com/site/about/guidelines.xhtml

Public, patient, and stakeholder involvement

We have worked closely with a stakeholder group in formulation of the proposal and have built in engagement as a key component to inform theory development. These stakeholders come from a range of relevant backgrounds, including patients and the public, heads of professional standards bodies, members of regulatory bodies and unions in the UK, influential theorists in the field, healthcare professionals with lived experience of unprofessional behaviours, and minority ethnic perspectives. This protocol incorporates four timepoints where stakeholder feedback will be incorporated (figure 1). This includes sense-checking our initial programme theories, aiding in identifying the most relevant evidence, ensuring our CMOCs reflect lived experience, and refining the final programme theories. In line with the ACTIVE (**A**uthors and **C**onsumers **T**ogether **I**mpacting on e**V**idencE) framework for stakeholder involvement in systematic reviews, our methodology is analogous to a continuous, multiple-time closed event approach with stakeholders able to influence the results of the review [44].

Stakeholder group feedback will be incorporated using the following processes to ensure rigour and relevance: 1) we will record what and how aspects of the developing theory are presented to stakeholders for refinement; 2) we will record where any alterations are suggested; 3) where suggestions are not aligned with what has been identified in the literature, we will perform purposive searching to sense-check recommendations; 4) any remaining discrepancies will be discussed within the team to determine majority consensus and 5) re-presentation of changes made in response to feedback to individual stakeholders or the whole group to further sense-check any changes [45]. We will take detailed notes of the meetings with the aid of a project administrator and will follow up with stakeholders individually regarding specific points raised.

Step 1: Identifying existing theories

To develop initial programme theories, we will iteratively:

a) Draw on preliminary discussions within the project team, with the healthcare workforce, patients, and the public;
b) Consult with our multidisciplinary stakeholder group as outlined above;
c) Examine healthcare literature already known to the research team (namely papers, reviews and reports identified in our initial scoping review which informed the funding proposal development), informal searching of key organisational websites (e.g., King's Fund, BMA, NHS England) and use of the 26 behaviours reported by Westbrook et al. for comparison against behaviours identified in the literature [2].

This first informal screening of the literature will sensitise the team to the breadth and depth of published and unpublished literature on unprofessional behaviours within healthcare. By investigating the theoretical underpinnings of programmes, we shall map the conceptual and theoretical landscape of unprofessional behaviour causes, outcomes, and how any identified strategies and interventions are theorised to work in acute healthcare settings. Building these initial programme theories will require iterative discussions within the project team and with our stakeholders to make sense of and synthesise the different assumptions. Once the programme theories have been developed by the project team, they will be presented to the stakeholder group to integrate their feedback and to ensure practical applicability (figure 1).

For peer review only - http://bmjopen.bmj.com/site/about/guidelines.xhtml

Step 2: Searching for evidence

Realist reviews typically draw on a combination of systematic searches, more informal, purposive searches, citation tracking and reference scanning [39]. It is also common to perform more searches later in the process in an evolutionary manner to further explain certain areas which are found to be underexplored in the programme theory. Additionally, in realist reviews, what is considered evidence often differs from other reviews. For example, non-empirical studies and commentaries can be included if they illuminate the topic being studied or can otherwise contribute to the programme theory. In realist reviews, the purpose is not to necessarily to identify every item of literature that could relate to a phenomenon, but, rather, to develop and refine a comprehensive theory to explain how the intervention works, why, for whom, and in what circumstances.

Search strategy

We will take a systematic approach and conduct searches to identify literature with which to test and refine our initial theories. In this formal, primary search we will:

a) Identify studies addressing strategies to reduce unprofessional behaviours in acute healthcare settings with all healthcare staff. Search academic databases including Medline, Embase and CINAHL and sources of trade, policy, and grey literature including Agency for Healthcare Research and Quality's Patient Safety Network (PSNET) and Google Scholar. Examples of trade journals we will search include AACN Bold Voices, Nursing Times, ED Management, and Nursing Standard. Search strategies will comprise search terms, synonyms, and index terms for: Acute care AND Healthcare staff AND Unprofessional behaviours. Searches from existing similar reviews such as Iling et al. (2013) will be consulted to aid identifying relevant search terms [36]. See supplementary file 1 for an indicative example search strategy, which is not complete, as searches are likely to evolve as is typical in a realist synthesis [39].
b) Conduct a grey literature search for professional codes of conduct produced by the Royal Colleges and the literature on cases brought to the Nursing and Midwifery Council, Health Care and Professions Council, and General Medical Council for unprofessional behaviour; using databases such as Health Management Information Consortium database and websites including NHS Employers, NHS Health Education England and Google (limited to screening the first 200 results).

Search strategies will be peer-reviewed using the PRESS (Peer Review of Electronic Search Strategies) method [46]. All search results will be saved in reference management software. A detailed, shared spreadsheet will record all searches conducted, to ensure transparency when reporting the search activities.

Additional searching

If we find that we require more data to develop, confirm, refute, or refine programme theory development, we will conduct additional purposive searches using CLUSTER search methods [47] to further develop any areas of the theory which require greater evidence to fully understand. These and earlier searches will follow the iterative realist search methods described by Booth et al. (2020) [48].

For peer review only

For peer review only - http://bmjopen.bmj.com/site/about/guidelines.xhtml

BMJ Open

Step 3: Article selection

Inclusion and exclusion criteria

We will include both peer-reviewed and grey literature which help understand how and why strategies to reduce unprofessional behaviours in acute care settings work and whom they benefit. The following inclusion criteria will be used (table 1).

Table 1. Inclusion criteria.

Category	Criterion
Study design	Any (including non-empirical papers/ reports).
Study setting	Acute healthcare settings - acute, critical, emergency (and potentially wider, see relevance criteria below).
Types of unprofessional behaviour	All as exhibited and experienced by healthcare staff (not patients nor patient to staff).
Types of participants	All employed staff groups including students on placements.
Types of interventions/strategies	Individual, team, organisational, and policy level interventions. Cyber-bullying and other forms of online unprofessional behaviour included if it is staff-to-staff only.
Causes of unprofessional behaviours	All.
Outcomes	Included but not limited to all sources that focused on one or more of the following aspects: staff wellbeing (stress, burnout, resilience) staff turnover, absenteeism, malpractice claims, patient complaints, magnet hospital/recruitment, patient safety (avoidable harm, errors, speaking up rates, safety incidents, improved listening/response), cost.
Language	Only sources in English will be included.

Searches will not be limited (e.g., by date or publication) but records tagged as ‘children’, ‘animals’ or ‘elder abuse’ will be removed, and we will seek to exclude papers describing staff to patient or patient to staff unprofessional behaviours.

Screening, relevancy, and rigour

Screening of search results will be primarily undertaken by JAA in collaboration with JM, MP or RA who will screen a subset of records retrieved. A 10% random sub-sample of the citations retrieved from searching will be reviewed independently for quality control (by a second reviewer, either JM, MP or RA) at title and abstract, full text, and relevancy stages. Any disagreements will be resolved by discussion between JA, the second reviewer and JM. If disagreements remain, then a third member of the team (AJ, RM or JMW) will review and any disagreements will be resolved through further review/discussion, with final decision

For peer review only - http://bmjopen.bmj.com/site/about/guidelines.xhtml

BMJ Open

resting with JM. The remaining 90% of decisions at these stages will be made by JAA. Screening of titles and abstracts will be performed using Rayyan.ai software (http://www.rayyan.ai/) and full texts will be screened using Mendeley (Mendeley Ltd.) [49].

Beyond the inclusion and exclusion criteria above, decisions regarding the inclusion or exclusion of sources will be based on a combination of relevance (based on both the major/minor criteria below and the ability to inform programme theories, i.e. conceptual richness and depth of sources) and rigour (whether the methods used to generate the relevant data are credible and trustworthy). Assessment of rigour will focus on the extent to which sources provide a detailed description of their methods and how generalisable and trustworthy their findings are based on those methods [39].

Our formal criteria for classifying the potential relevancy of sources are, by ascending order of relevancy:

Major contribution:

- Sources which contribute to the study aims and are conducted in an NHS context in acute care; or,
- Sources which contribute to the study aims and are conducted in an NHS context; or,
- Sources which contribute to the study aims and are conducted in contexts with similarities to the NHS (e.g., universal, publicly-funded health-care systems);

Minor contribution:

- Sources conducted in non-UK health-care systems that are markedly different to the NHS (e.g., fee-for-service, private insurance scheme systems) but where the mechanisms causing or moderating unprofessional behaviours could plausibly operate in the context of those working in the NHS; or,
- Sources which contribute to the study aims and can clearly help to identify mechanisms which could plausibly operate in the context of the NHS (e.g., law, police, and army).

We will prioritise the papers of major relevance in relation to the above criteria. These sources will then be sorted into the above categories in a reference manager and assessed for their ability to inform the refinement of programme theories (theoretical relevancy and conceptual richness). If we are unable to develop and refine aspects of the programme theory due to scarcity in majorly relevant sources, we will then draw on literature from the minor relevancy criteria above.

Step 4: Data extraction

In keeping with a realist retroductive analysis, relevant sections of texts that have been interpreted as related to contexts, mechanisms and/or their relationships to outcomes will be coded and organised in NVivo 12 software (QSR International) . This coding will be both inductive (codes created to categorise data reported in included sources) and deductive (codes created in advance of data extraction and analysis as informed by the initial programme theory). Each new element of relevant data will be used to test and refine aspects of the programme theory, and as it is refined, included sources will be re-scrutinised to search for data relevant to the revised programme theory that may have been missed

For peer review only - http://bmjopen.bmj.com/site/about/guidelines.xhtml

initially. The characteristics of the included sources will be extracted separately into an Excel spreadsheet to provide a descriptive overview of (but not limited to) settings, intervention, participants, and context.

As outlined above, we will start the coding and analysis process using the literature that has been deemed to make a 'major' i.e., most relevant contribution to the research questions to start building and refining our programme theory, while progressively focusing the review. Articles categorised as providing 'minor' contributions will be held back and analysed if there is no 'major' study which sheds light on certain aspects of the programme theory. The aim of the review will be to reach theoretical saturation in relation to the objectives, rather than to aggregate every single study that exists in the area. All study-related decisions will be documented and recorded as part of an audit trail to increase transparency and ensure consistency and rigour.

Step 5: Synthesising evidence and drawing conclusions

Our data analysis will use realist logic to make sense of the initial programme theory. Data will be interrogated at individual, team, and organisational levels to establish their relationships. This type of analysis will enable us to understand how the most relevant and important mechanisms work in different contexts, thus, allowing us to build more transferable CMOCs – these will be fully formulated by the end of this step. During the review, we will move iteratively between the analysis of particular examples from the literature, refinement of programme theory, and further iterative searching for data to test particular subsections of the programme theory as required (figure 1). We will also use the following strategies to make sense of the data (table 2) [50,51].

Table 2. Strategies foundational to the realist analysis.

**Strategies for synthesising evidence**
Compare and contrast sources of evidence – for example, where evidence about interventions or its mechanisms in one source allows insights into evidence about outcomes in another paper.
Reconciling of sources of evidence – where results differ in apparently similar circumstances, further investigation is appropriate in order to find explanations for why these different results occurred.
Adjudication of sources of evidence – this will enable us to make the synthesis more manageable. This includes strategies outlined in 'article selection' section above, i.e. dividing papers that make 'major' or 'minor' contributions to our research questions [51].
Consolidation of sources of evidence – where outcomes differ in particular contexts, an explanation can be constructed of how and why these outcomes occur differently.

Many of these processes are analogous to the identification of demi-regularities (*"semi-predictable patterns or pathways of programme functioning"* across studies). This type of thinking will be essential to help identify underlying mechanisms that are common to different strategies [40,52]. As required, we will also identify and incorporate middle range theories (e.g., theories around, for example, groupthink and psychological safety) to enable us to move beyond description and understand the 'set of assumptions' underlying the observed associations [53].

For peer review only - http://bmjopen.bmj.com/site/about/guidelines.xhtml

Step 6: Testing and refining, and developing resources for stakeholders

We will then test and refine our programme theories with our stakeholder group. Informed by the 'Evidence Integration Triangle' [54] and stakeholder involvement at this final timepoint, we will use findings from our realist review to produce actionable evidence to support NHS managers/leaders to better understand how work environments may help or hinder unprofessional behaviours and identify what strategies work where.

Ethics and dissemination

Dissemination

Dissemination activities comprise step 7 of our project. The project will produce six major types of output in collaboration with our stakeholder group and will present some of these at our end of study project dissemination workshop (table 3).

Table 3. Dissemination and pathways to impact and who is reached [55].

Output	Description	Timescale to benefit	How will impact be achieved?	Who is reached?
**PPI / Stakeholder Engagement.**	PPI and our stakeholder representatives will be actively involved in the production of all outputs. The stakeholder group, including healthcare staff and PPI representatives, will be encouraged to think about alternative or additional approaches to dissemination.	During project and up to 6 months after.	Engaging with the stakeholder group will enable us to understand how best to reach people like them in the most well-targeted manner.	Internal stakeholder group of study and their immediate contacts.
**Media engagement strategy.**	We will identify the most appropriate way to engage with our non-Academic stakeholder groups. For example, through engagement with relevant professional bodies (e.g., British Medical Association, General Medical Council, Royal College of Nursing, Royal College of Midwives, NHS Employers etc) and through promoting our findings via alternative publication routes (e.g., Health Services Journal, Nursing Times/Standard, BMJ, The Conversation, Twitter).	During project and up to 2 years after.	Engaging with media will allow lighter touch but wider dissemination of our key messages.	Public, academics, practitioners, managers, leaders, policymakers.

For peer review only - http://bmjopen.bmj.com/site/about/guidelines.xhtml

**Resources for NHS managers/leaders.**	Tailored resources for NHS managers/leaders, and organisations supporting the healthcare workforce regarding how to implement strategies to reduce unprofessional behaviours.	End of project onwards.	Effects will be visible once stakeholders are able to implement changes and evaluate the impact of those changes.	Managers, leaders.
**Plain English summaries.**	We will create plain English summaries tailored to different audiences.	End of project and up to 2 years thereafter.	This will achieve impact through knowledge transfer in the short- to medium-term (1 month-2 years).	Healthcare professionals, managers, leaders, training providers, policymakers.
**Innovative forms of communication.**	We have had positive experiences of involving film makers and using the medium of theatre to perform research findings [56].	1-5 years.	Such outputs will create a longer-lasting impression with stakeholders than other more traditional forms of dissemination.	Healthcare professionals, managers, leaders, training providers, policymakers.
**Academic outputs.**	In addition to the main project report, an overall findings paper will be submitted to a high-impact peer-reviewed journal, and conference presentations at healthcare staff wellbeing conferences (such as Health Services Research UK) will be pursued.	3-5 years.	Informing the agenda for debate and action in health services and in public policy more widely.	Academics, managers, policymakers.

Ethical approval

The University of Surrey's Ethics committee have confirmed that ethical review is not needed for this realist review of secondary evidence.

Author contributions

JM led the conceptualisation and development of the overall study design and drafting of the protocol on which this paper is based. JAA drafted the manuscript in dialogue with JM and all authors and helped to refine the communication of the study methodology. RA, MP, JMW, AJ, RM and JW all contributed to the study conceptualisation and design of the study protocol. All authors substantively revised drafts of this manuscript and approved the final version.

For peer review only - http://bmjopen.bmj.com/site/about/guidelines.xhtml

Funding statement

This project was supported by the NIHR HS&DR Programme with grant number 131606. The views and opinions expressed herein are those of the authors and do not necessarily reflect those of the HS&DR programme.

Competing interests statement

None declared.

Patient consent

Not required.

References

1 Katz D, Blasius K, Isaak R, *et al.* Exposure to incivility hinders clinical performance in a simulated operative crisis. *BMJ Qual Saf* 2019;**28**:750–7. doi:10.1136/bmjqs-2019-009598

2 Westbrook J, Sunderland N, Li L, *et al.* The prevalence and impact of unprofessional behaviour among hospital workers: a survey in seven Australian hospitals. *Med J Aust* 2021;**214**:31–7. doi:10.5694/mja2.50849

3 Carter M, Thompson N, Crampton P, *et al.* Workplace bullying in the UK NHS: A questionnaire and interview study on prevalence, impact and barriers to reporting. *BMJ Open* 2013;**3**. doi:10.1136/bmjopen-2013-002628

4 Jones A, Blake J, Banks C, *et al.* Speaking up about Bullying and Harassment in Healthcare: Reflections Following the Introduction of an Innovative "Speak Up" Role in NHS England. In: *Connecting Healthcare Worker Well-Being, Patient Safety and Organisational Change*. Springer 2020. 145–61.

5 Felblinger DM. Bullying, incivility, and disruptive behaviors in the healthcare setting: identification, impact, and intervention. *Front Health Serv Manage* 2009;**25**:13–23. doi:10.1097/01974520-200904000-00003

6 Cruz D, Rodriguez Y, Mastropaolo C. Perceived microaggressions in health care: A measurement study. *PLoS One* 2019;**14**:1–11. doi:10.1371/journal.pone.0211620

7 Westbrook J, Sunderland N, Atkinson V, *et al.* Endemic unprofessional behaviour in health care: the mandate for a change in approach. *Med J Aust* 2018;**209**:380–1. doi:10.5694/mja17.01261

8 NHS England. NHS staff survey 2020. 2021. https://www.nhsstaffsurveys.com/

9 NHS Equality and Diversity Council. WRES 2018. Published Online First: 2018.https://www.england.nhs.uk/wp-content/uploads/2018/12/wres-2018-report-v1.pdf

10 Sue DW. *Microaggressions in everyday life: Race, gender, and sexual orientation*. John Wiley & Sons 2010.

11 Wild JRL, Ferguson HJM, McDermott FD, *et al.* Undermining and bullying in surgical training: A review and recommendations by the Association of Surgeons in Training. *Int J Surg* 2015;**23**:S5–9. doi:10.1016/j.ijsu.2015.09.017

For peer review only - http://bmjopen.bmj.com/site/about/guidelines.xhtml

For peer review only

12 Pisklakov S, Tilak V, Patel A, *et al.* Bullying and Aggressive Behavior among Health Care Providers: Literature Review. *Adv Anthropol* 2013;**03**:179–82. doi:10.4236/aa.2013.34024

13 Ariza-Montes A, Muniz NM, Montero-Simó MJ, *et al.* Workplace bullying among healthcare workers. *Int J Environ Res Public Health* 2013;**10**:3121–39. doi:10.3390/ijerph10083121

14 Kline R, Lewis D. The price of fear: Estimating the financial cost of bullying and harassment to the NHS in England. *Public Money Manag* 2019;**39**:166–74. doi:10.1080/09540962.2018.1535044

15 Mannion R, Davies H. Raising and responding to frontline concerns in healthcare. *BMJ* 2019;**366**:1–4. doi:10.1136/bmj.l4944

16 Rosenstein AH. Nurse-physician relationships: Impact on nurse satisfaction and retention. *Am J Nurs* 2002;**102**:26–34. doi:10.1097/00000446-200206000-00040

17 NHS Employers. Tackling bullying & harassment in the NHS. Tackling bullying NHS infographic. 2019.https://www.nhsemployers.org/articles/tackling-bullying-nhs-infographic

18 Lever I, Dyball D, Greenberg N, *et al.* Health consequences of bullying in the healthcare workplace: A systematic review. *J Adv Nurs* 2019;**75**:3195–209. doi:10.1111/jan.13986

19 Nielsen MB, Indregard A-MR, Overland S. Workplace bullying and sickness absence: a systematic review and meta-analysis of the research literature. 2016.

20 Edmondson A. Psychological safety and learning behavior in work teams. 1999;**44**:350–83.https://doi.org/10.2307/2666999

21 Edmondson A. The Role of Psychological Safety. 2019. doi:10.1002/ltl.20419

22 Martinez W, Lehmann LS, Thomas EJ, *et al.* Speaking up about traditional and professionalism-related patient safety threats: A national survey of interns and residents. *BMJ Qual Saf* 2017;**26**:869–80. doi:10.1136/bmjqs-2016-006284

23 Barzallo Salazar MJ, Minkoff H, Bayya J, *et al.* Influence of surgeon behavior on trainee willingness to speak up: A randomized controlled trial. *J Am Coll Surg* 2014;**219**:1001–7. doi:10.1016/j.jamcollsurg.2014.07.933

24 Riskin A, Erez A, Foulk TA, *et al.* Rudeness and medical team performance. *Pediatrics* 2017;**139**. doi:10.1542/peds.2016-2305

25 Riskin A, Erez A, Foulk TA, *et al.* The impact of rudeness on medical team performance: A randomized trial. *Pediatrics* 2015;**136**:487–95. doi:10.1542/peds.2015-1385

26 Cooper WO, Spain DA, Guillamondegui O, *et al.* Association of Coworker Reports about Unprofessional Behavior by Surgeons with Surgical Complications in Their Patients. *JAMA Surg* 2019;**154**:828–34. doi:10.1001/jamasurg.2019.1738

27 Schoville R, Aebersold M. How Workplace Bullying and Incivility Impacts Patient Safety: A Qualitative Simulation Study Using BSN Students. *Clin Simul Nurs* 2020;**45**:16–23. doi:10.1016/j.ecns.2020.01.003

28 Doran N, Fox F, Rodham K, *et al.* Lost to the NHS: A mixed methods study of why GPS leave practice early in England. *Br J Gen Pract* 2016;**66**:e128–34. doi:10.3399/bjgp16X683425

29 Hogh A, Mikkelsen E, Hansen Å. Individual Consequences of Workplace Bullying/Mobbing. In: *Bullying and Harassment in the Workplace*. 2010. 107–28. doi:10.1201/ebk1439804896-7

For peer review only - http://bmjopen.bmj.com/site/about/guidelines.xhtml

30 Woodrow C, Guest D. A comparison of factors associated with workplace bullying in three UK Healthcare Organisations. In: *New Challenges for a Healthy Workplace in Human Services*. Rainer Hampp Verlag 2011. 87–104.

31 British Medical Association. Workplace bullying and harassment of doctors A review of recent research. 2017. file:///Users/VWC/Downloads/Bullying and harassment research review v7 WEB.pdf

32 Armstrong N. Management of Nursing Workplace Incivility in the Health Care Settings: A Systematic Review. *Work Heal Saf* 2018;**66**:403–10. doi:10.1177/2165079918771106

33 Gillen PA, Sinclair M, Kernohan WG, *et al.* Interventions for prevention of bullying in the workplace (Review). *Cochrane Database Syst Rev* 2017;:12. doi:10.1002/14651858.CD009778.pub2

34 Mannion R, Davies H, Powell M, *et al.* Healthcare scandals and the failings of doctors: Do official inquiries hold the profession to account? *J Health Organ Manag* 2019;**33**:221–40. doi:10.1108/JHOM-04-2018-0126

35 Keller S, Yule S, Zagarese V, *et al.* Predictors and triggers of incivility within healthcare teams: A systematic review of the literature. *BMJ Open* 2020;**10**:1–15. doi:10.1136/bmjopen-2019-035471

36 Illing J, Carter M, Thompson NJ, *et al.* Evidence synthesis on the occurrence, causes , management of bullying and harassing behaviours to inform decision making in the NHS. 2013;**44**:54–168.

37 Raine G, Thomas S, Rodgers M, *et al. Workplace-based interventions to promote healthy lifestyles in the NHS workforce: a rapid scoping and evidence map*. 2020. doi:10.3310/hsdr08180

38 Pawson R, Greenhalgh T, Harvey G, *et al.* Realist review - A new method of systematic review designed for complex policy interventions. *J Heal Serv Res Policy* 2005;**10**:21–34. doi:10.1258/1355819054308530

39 Wong G, Westhorp G, Pawson R, *et al.* Realist Synthesis: RAMESES Training Materials. *RAMESES Proj* 2013;:55.

40 Dalkin SM, Greenhalgh J, Jones D, *et al.* What's in a mechanism? Development of a key concept in realist evaluation. 2015;**10**:49.https://doi.org/10.1186/s13012-015-0237-x

41 Greenhalgh J, Manzano A. Understanding 'context' in realist evaluation and synthesis. *Int J Soc Res Methodol* 2021;**00**:1–13. doi:10.1080/13645579.2021.1918484

42 Sayer RA. *Method in social science: A realist approach*. Psychology Press 1992.

43 Wong G, Greenhalgh T, Westhorp G, *et al.* RAMESES publication standards: realist syntheses. *BMC Med* 2013;**11**:1–14.

44 Pollock A, Campbell P, Struthers C, *et al.* Development of the ACTIVE framework to describe stakeholder involvement in systematic reviews. *J Heal Serv Res Policy* 2019;**24**:245–55. doi:10.1177/1355819619841647

45 Abrams R, Park S, Wong G, *et al.* Lost in reviews: Looking for the involvement of stakeholders, patients, public and other non-researcher contributors in realist reviews. *Res Synth Methods* 2021;**12**:239–47. doi:10.1002/jrsm.1459

46 McGowan J, Sampson M, Salzwedel DM, *et al.* PRESS Peer Review of Electronic Search Strategies: 2015 Guideline Statement. *J Clin Epidemiol* 2016;**75**:40–6. doi:10.1016/j.jclinepi.2016.01.021

47 Booth A, Harris J, Croot E, *et al.* Towards a methodology for cluster searching to

For peer review only - http://bmjopen.bmj.com/site/about/guidelines.xhtml

BMJ Open

provide conceptual and contextual "richness" for systematic reviews of complex interventions: case study (CLUSTER). *BMC Med Res Methodol* 2013;**13**:1–14.

48 Booth A, Briscoe S, Wright JM. The "realist search": A systematic scoping review of current practice and reporting. *Res Synth Methods* 2020;**11**:14–35.

49 Ouzzani M, Hammady H, Fedorowicz Z, *et al.* Rayyan-a web and mobile app for systematic reviews. *Syst Rev* 2016;**5**:1–10. doi:10.1186/s13643-016-0384-4

50 Pawson R. *Evidence-based policy: a realist perspective*. sage 2006.

51 Pearson M, Chilton R, Wyatt K, *et al.* Implementing health promotion programmes in schools: A realist systematic review of research and experience in the United Kingdom. *Implement Sci* 2015;**10**:1–20. doi:10.1186/s13012-015-0338-6

52 Gilmore B, McAuliffe E, Power J, *et al.* Data Analysis and Synthesis Within a Realist Evaluation: Toward More Transparent Methodological Approaches. *Int J Qual Methods* 2019;**18**:1–11. doi:10.1177/1609406919859754

53 Merton RK. On theoretical sociology: five essays, old and new. 1967.

54 Glasgow RE, Green LW, Taylor M V., *et al.* An evidence integration triangle for aligning science with policy and practice. *Am J Prev Med* 2012;**42**:646–54. doi:10.1016/j.amepre.2012.02.016

55 Lamont T. *Making Research Matter: Steps to Impact for Health and Care Researchers*. Policy Press 2021.

56 Maben J, Taylor C, Dawson J, *et al.* A realist informed mixed-methods evaluation of Schwartz Center Rounds® in England. *Heal Serv Deliv Res* 2018;**6**:1–260. doi:10.3310/hsdr06370

For peer review only

For peer review only - http://bmjopen.bmj.com/site/about/guidelines.xhtml

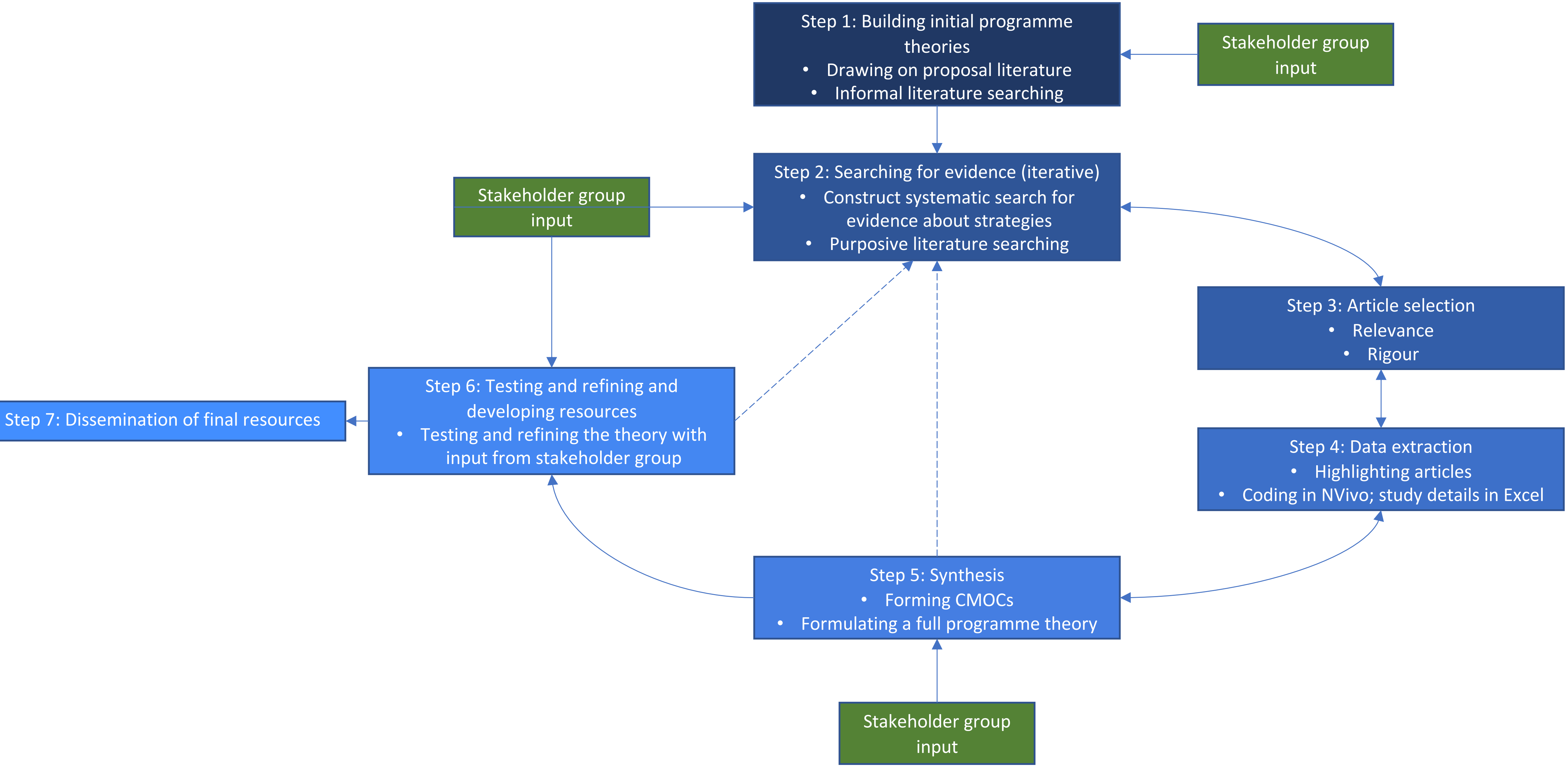
Step 1: Building initial programme theories
• Drawing on proposal literature
• Informal literature searching
Stakeholder group input
Stakeholder group input
Step 2: Searching for evidence (iterative)
• Construct systematic search for evidence about strategies
• Purposive literature searching
Step 3: Article selection
• Relevance
• Rigour
Step 7: Dissemination of final resources
Step 6: Testing and refining and developing resources
• Testing and refining the theory with input from stakeholder group
Step 4: Data extraction
• Highlighting articles
• Coding in NVivo; study details in Excel
Step 5: Synthesis
• Forming CMOCs
• Formulating a full programme theory
Stakeholder group input

Literature search strategy examples

Unprofessional Behaviours among Acute Care staff – MEDLINE systematic strategy (draft)

1. Ovid MEDLINE(R) ALL <1946 to February 01, 2022>

1 emergency medical services/ or advanced trauma life support care/ or call centers/ or emergency medical dispatch/ or emergency medical service communication systems/ 47376

2 exp airway management/ or exp emergency treatment/ or exp ambulatory care/ or exp critical care/ or exp perioperative care/ or exp preoperative care/ 456732

3 exp emergency service, hospital/ or emergency services, psychiatric/ or hotlines/ or poison control centers/ or exp "transportation of patients"/ or triage/ or critical care nursing/ or emergency nursing/ 129117

4 (emergenc* adj5 (care or service* or health* or ill* or treat* or medic* or unit* or centre* or centre* or department* or setting*)).tw,kf. 200510

5 (acute* adj5 (care or service* or health* or ill* or treat* or medic* or unit* or centre* or centre* or department* or setting* or ward?)).tw,kf. 220374

6 (trauma* adj5 (care or service* or ill* or unit* or centre* or centre* or department*)).tw,kf. 26629

7 (ambula* adj5 (care or service* or unit* or centre* or centre* or department* or setting*)).tw,kf. 26105

8 (critical* adj2 (care or ill*)).tw,kf. 92228

9 (urgent adj3 (care or service* or medic*)).tw,kf. 5298

10 "intensive care".tw,kf. 171169

11 or/1-10 [Acute Care or Ambulance services] 1058529

12 agonistic behavior/ 1778

13 exp bullying/ 5615

14 problem behavior/ 3232

15 exp harassment, non-sexual/ 5697

16 sexual harassment/ 2020

17 incivility/ 232

18 Professional Misconduct/ 3432

19 Hostility/ 5396

20 Social Discrimination/ 1494

21 bully*.tw,kf. 7035

22 harass*.tw,kf. 4646

For peer review only

23 intimidat*.tw,kf. 1506

24 (lateral* adj2 violence).tw,kf. 100

25 (horizontal* adj2 violence).tw,kf. 146

26 (transgressive adj3 behavio?r*).tw,kf. 39

27 (disruptive adj3 behavio?r*).tw,kf. 4362

28 (unprofessional adj3 behavio?r*).tw,kf. 399

29 (micro-aggress* or microaggress*).tw,kf. 474

30 incivil*.tw,kf. 683

31 uncivil*.tw,kf. 212

32 rude*.tw,kf. 1193

33 mistreat*.tw,kf. 2441

34 (professional* adj3 misconduct).tw,kf. 303

35 mobbing.tw,kf. 416

36 (negative behavio* or negative act?).tw,kf. 1713

37 or/12-36 [Unprofessional behaviours] 40104

38 exp Health Personnel/ 571903

39 exp Students, Health Occupations/ 79772

40 exp education, graduate/ or "internship and residency"/ or teaching rounds/ 94345

41 exp Interprofessional Relations/ 71796

42 Workplace/ 26778

43 (nurs* or midwif* or midwiv*).tw,kf. 516310

44 paramedic?.tw,kf. 6034

45 (doctor? or physician? or clinician? or surgeon? or consultant?).tw,kf. 1002988

46 (student? adj2 (medic* or health* or clinic*)).tw,kf. 65732

47 intern?.tw,kf. 11160

48 resident?.tw,kf. 178976

49 (Therapist? or Pharmacist? or Optometrist? or Nutritionist? or Dentist? or Physiotherapist?).tw,kf. 132472

50 (Audiologist? or Anatomist? or Allergist? or An?esthetist? or An?esthesiologist? or Cardiologist? or Endocrinologist? or Gastroenterologist? or GP? or Geriatrician? or Hospitalist? or Oncologist?).tw,kf. 225380

For peer review only

51 (Ophthalmologist? or Otolaryngologist? or Pathologist? or P?ediatrician? or Physiatrist? or Psychiatrist? or Pulmonologist? or Radiographer? or Radiologist?).tw,kf. 170433

52 medic?.tw,kf. 21462

53 assistant?.tw,kf. 28781

54 (cleaner? or ancillary or porter?).tw,kf. 24901

55 (auxillary or auxillaries or administrator? or secretary or secretaries or receptionist? or technician?).tw,kf. 41498

56 (employee? or worker? or Staff or personnel or practitioner? or professional? or workforce*).tw,kf. 917727

57 (workplace* or "work place*" or worksite* or "work site*" or "work setting*").tw,kf. 55952

58 or/38-57 [Staff] 2959526

59 37 and 58 [UB and Staff search 1] 14614

60 physician-nurse relations/ or interprofessional relations/ 54899

61 aggression/ or prejudice/ or ageism/ or gender equity/ or homophobia/ or racism/ or sexism/ or weight prejudice/ or xenophobia/ 69260

62 60 and 61 [Aggression or prejudice among staff MeSH] 740

63 ((staff or employee* or work* or nurs* or doctor?) adj8 (WPV or violen*) adj5 among*).tw,kf. 560

64 ((staff or employee* or work* or nurs* or doctor?) adj8 Victim* adj5 among*).tw,kf. 109

65 ((staff or employee* or work* or nurs* or doctor?) adj8 hostil*).tw,kf. 497

66 ((staff or employee* or work* or nurs* or doctor?) adj3 undermin*).tw,kf. 334

67 ((staff or employee* or work* or nurs* or doctor?) adj6 discriminat*).tw,kf. 3201

68 "abusive supervision".tw,kf. 140

69 (workplace adj3 conflict*).tw,kf.174

70 ((staff or employee* or work* or nurs* or doctor?) adj5 (gender adj2 (inequalit* or equalit* or discriminat*))).tw,kf. 281

71 ((staff or employee* or work* or nurs* or doctor?) adj5 (racism or racist or (racial adj3 abus*))).tw,kf. 330

72 ((staff or employee* or work* or nurs* or doctor?) adj5 (sexism or sexist)).tw,kf. 83

73 ((staff or employee* or work* or nurs* or doctor?) adj8 ((disabilit* or disabled) adj5 (inequalit* or equalit* or discriminat*))).tw,kf. 97

74 ((staff or employee* or work* or nurs* or doctor?) adj8 ableis*).tw,kf. 6

75 or/62-74 [UB and staff search 2]6234

For peer review only

76 59 or 75 [UB among Staff final search] 20009

77 11 and 76 [UB among Staff in Acute Care] 960

78 afghanistan/ or africa/ or africa, northern/ or africa, central/ or africa, eastern/ or "africa south of the sahara"/ or africa, southern/ or africa, western/ or albania/ or algeria/ or andorra/ or angola/ or "antigua and barbuda"/ or argentina/ or armenia/ or azerbaijan/ or bahamas/ or bahrain/ or bangladesh/ or barbados/ or belize/ or benin/ or bhutan/ or bolivia/ or borneo/ or "bosnia and herzegovina"/ or botswana/ or brazil/ or brunei/ or bulgaria/ or burkina faso/ or burundi/ or cabo verde/ or cambodia/ or cameroon/ or central african republic/ or chad/ or exp china/ or comoros/ or congo/ or cote d'ivoire/ or croatia/ or cuba/ or "democratic republic of the congo"/ or cyprus/ or djibouti/ or dominica/ or dominican republic/ or ecuador/ or egypt/ or el salvador/ or equatorial guinea/ or eritrea/ or eswatini/ or ethiopia/ or fiji/ or gabon/ or gambia/ or "georgia (republic)"/ or ghana/ or grenada/ or guatemala/ or guinea/ or guinea-bissau/ or guyana/ or haiti/ or honduras/ or independent state of samoa/ or exp india/ or indian ocean islands/ or indochina/ or indonesia/ or iran/ or iraq/ or jamaica/ or jordan/ or kazakhstan/ or kenya/ or kosovo/ or kuwait/ or kyrgyzstan/ or laos/ or lebanon/ or liechtenstein/ or lesotho/ or liberia/ or libya/ or madagascar/ or malaysia/ or malawi/ or mali/ or malta/ or mauritania/ or mauritius/ or mekong valley/ or melanesia/ or micronesia/ or monaco/ or mongolia/ or montenegro/ or morocco/ or mozambique/ or myanmar/ or namibia/ or nepal/ or nicaragua/ or niger/ or nigeria/ or oman/ or pakistan/ or palau/ or exp panama/ or papua new guinea/ or paraguay/ or peru/ or philippines/ or qatar/ or "republic of belarus"/ or "republic of north macedonia"/ or romania/ or exp russia/ or rwanda/ or "saint kitts and nevis"/ or saint lucia/ or "saint vincent and the grenadines"/ or "sao tome and principe"/ or saudi arabia/ or serbia/ or sierra leone/ or senegal/ or seychelles/ or singapore/ or somalia/ or south africa/ or south sudan/ or sri lanka/ or sudan/ or suriname/ or syria/ or taiwan/ or tajikistan/ or tanzania/ or thailand/ or timor-leste/ or togo/ or tonga/ or "trinidad and tobago"/ or tunisia/ or turkmenistan/ or uganda/ or ukraine/ or united arab emirates/ or uruguay/ or uzbekistan/ or vanuatu/ or venezuela/ or vietnam/ or west indies/ or yemen/ or zambia/ or zimbabwe/ 1196785

79 "Organisation for Economic Co-Operation and Development"/ 413

80 australasia/ or exp australia/ or austria/ or baltic states/ or belgium/ or exp canada/ or chile/ or colombia/ or costa rica/ or czech republic/ or exp denmark/ or estonia/ or europe/ or finland/ or exp france/ or exp germany/ or greece/ or hungary/ or iceland/ or ireland/ or israel/ or exp italy/ or exp japan/ or korea/ or latvia/ or lithuania/ or luxembourg/ or mexico/ or netherlands/ or new zealand/ or north america/ or exp norway/ or poland/ or portugal/ or exp "republic of korea"/ or "scandinavian and nordic countries"/ or slovakia/ or slovenia/ or spain/ or sweden/ or switzerland/ or turkey/ or exp united kingdom/ or exp united states/ 3378869

81 European Union/ 17082

82 Developed Countries/ 21055

83 or/79-82 3394093

84 78 not 83 [OECD search filter NICE 2021] 1110107

85 77 not 84 [UB among Acute Care Staff with non-OECD countries removed] 872

86 (exp Child/ or Adolescent/ or exp Infant/) not exp Adult/2018609

87 85 not 86 [Child studies removed] 804

For peer review only

88	(elder mistreat* or elder abuse* or elder neglect*).tw,kf.	1880
89	87 not 88 [Elder abuse studies removed]	780

Google Scholar search strategy examples

Google Scholar - Theories
Search 1
bullying | harassment | discrimination|unprofessional AND workplace|worker|staff AND model|framework|concept|idea|opinion|theory|view|perception|attitude|theories [Searched in Publish or Perish Title Field]

Search 2 – Interventions & strategies
interventions|strategies|techniques|program|programs|programme|programmes AND unprofessional|bullying|harassment|discrimination AND nurse|doctor|paramedic|hospital|ambulance|staff|professional AND emergency|acute|trauma [searched in Publish or Perish Keywords field]

For peer review only

	Reporting item	Description of item	Reported on page(s)
**Title**			
1		In the title, identify the document as a realist synthesis or review	1
**Abstract**			
2		While acknowledging publication requirements and house style, abstracts should ideally contain brief details of: the study's background, review question or objectives; search strategy; methods of selection, appraisal, analysis and synthesis of sources; main results; and implications for practice	1-2
**Introduction**			
3	Rationale for review	Explain why the review is needed and what it is likely to contribute to existing understanding of the topic area	4-5
4	Objectives and focus of review	State the objective(s) of the review and/or the review question(s). Define and provide a rationale for the focus of the review	5-6
**Methods**			

	Reporting item	Description of item	Reported on page(s)
5	Changes in the review process	Any changes made to the review process that was initially planned should be briefly described and justified	Potential changes outlined on pg 10-12
6	Rationale for using realist synthesis	Explain why realist synthesis was considered the most appropriate method to use	4
7	Scoping the literature	Describe and justify the initial process of exploratory scoping of the literature	8
8	Searching processes	While considering specific requirements of the journal or other publication outlet, state and provide a rationale for how the iterative searching was done. Provide details on all of the sources accessed for information in the review. Where searching in electronic databases has taken place, the details should include, for example, name of database, search terms, dates of coverage and date last searched. If individuals familiar with the relevant literature and/or topic area were contacted, indicate how they were identified and selected	9-10
9	Selection and appraisal of documents	Explain how judgements were made about including and excluding data from documents, and justify these	10-11
10	Data extraction	Describe and explain which data or information were extracted from the included documents and justify this selection	11-12

For peer review only

Reporting item		Description of item	Reported on page(s)
11	Analysis and synthesis processes	Describe the analysis and synthesis processes in detail. This section should include information on the constructs analysed and describe the analytic process	12-13
**Results**			
12	Document flow diagram	Provide details on the number of documents assessed for eligibility and included in the review, with reasons for exclusion at each stage, as well as an indication of their source of origin (e.g. from searching databases, reference lists and so on). You may consider using the example templates (which are likely to need modification to suit the data) that are provided	7
13	Document characteristics	Provide information on the characteristics of the documents included in the review	n/a
14	Main findings	Present the key findings with a specific focus on theory building and testing	n/a
**Discussion**			
15	Summary of findings	Summarise the main findings, taking into account the reviews objective(s), research question(s), focus and intended audience(s)	n/a

For peer review only - http://bmjopen.bmj.com/site/about/guidelines.xhtml

Reporting item		Description of item	Reported on page(s)
16	Strengths, limitations and future research directions	Discuss both the strengths of the review and its limitations. These should include (but need not be restricted to) (a) consideration of all the steps in the review process and (b) comment on the overall strength of evidence supporting the explanatory insights which emerged<br>The limitations identified may point to areas where further work is needed	2 & 14
17	Comparison with existing literature	Where applicable, compare and contrast the reviews findings with the existing literature (e.g. other reviews) on the same topic	2-5
18	Conclusion and recommendations	List the main implications of the findings and place these in the context of other relevant literature. If appropriate, offer recommendations for policy and practice	14
19	Funding	Provide details of funding source (if any) for the review, the role played by the funder (if any) and any conflicts of interests of the reviewers	15

For peer review only
